# Synthetic target trial emulation and predictive modeling of amylin-pathway therapies for obesity and type 2 diabetes

**DOI:** 10.1016/j.metop.2025.100414

**Published:** 2025-10-31

**Authors:** Faisal A. Al-Harbi, Ahmed K. Alsaif, Atheer G. Almutairi, Hussam J. Alshehri, Elan A. Aleidan, Ghaida S. Alabdulaaly, Mashael E. Alanazi, Ahmed Y. Azzam

**Affiliations:** a College of Medicine, Qassim University, Qassim, Saudi Arabia; b College of Medicine, Al-Rayan Colleges, Al-Madinah, Saudi Arabia; c College of Medicine, King Abdulaziz University, Jeddah, Saudi Arabia; d Department of Internal Medicine, King Fahad Medical City, Riyadh, Saudi Arabia; e Director of Clinical Research and Clinical Artificial Intelligence, ASIDE Healthcare, Lewes, DE, USA; f Division of Global Health and Public Health, School of Nursing, Midwifery and Public Health, University of Suffolk, Ipswich, Suffolk, United Kingdom

**Keywords:** Amylin, Amycretin, Diabetes, Obesity, Target trial emulation

## Abstract

**Introduction:**

Amylin-pathway therapies represent a novel therapeutic class for obesity and type 2 diabetes, however head-to-head comparative data and long-term outcome predictions remain limited. We conducted target trial emulation and computational predictive modeling aiming to predict future trial outcomes and comparative effectiveness across the amylin-pathway development program.

**Methods:**

Following PRISMA 2020 and TARGET framework guidelines, we search in the current literature for eligible trials and extracted data from seven randomized controlled trials (N = 5,786 participants) of amylin-pathway therapies published up to September 2025. We reconstructed high-precision synthetic individual patient data (IPD) and developed computational models for virtual head-to-head comparisons, dose-response optimization, longitudinal trajectory prediction, and trial simulation. Network meta-analysis integrated evidence across CagriSema, cagrilintide, and amycretin formulations.

**Results:**

Synthetic IPD reconstruction achieved >99 % fidelity to source trials, validated through leave-trial-out cross-validation (efficacy RMSE: 2.9 % points, calibration slope: 0.61; discontinuation RMSE: 0.18, slope: 1.08). Virtual head-to-head modeling confirmed CagriSema superiority over amycretin subcutaneous at matched timepoints (posterior probability >0.95). Dose-response modeling identified optimal amycretin exposures (ED80: 8.88 mg subcutaneous, 95 % CI: 7.12–11.08), with benefit-risk frontier analysis delineating a therapeutic window at 10–20 mg balancing efficacy plateau against tolerability thresholds (GI-AE <75 %, discontinuation <20 %). Longitudinal kinetics showed plateau timing at 52–68 weeks for obesity outcomes and 24–32 weeks for glycemic endpoints. Heterogeneity analysis revealed complete resolution for GI adverse events (I^2^_DL = 0 %, τ^2^ = 0) and moderate variation for discontinuation (I^2^_DL = 13 %, τ^2^ = 0.03) after logit-scale correction with proper within-arm variance weighting. Machine learning models predicted treatment response with 82–87 % accuracy using baseline characteristics.

**Conclusions:**

Synthetic target trial emulation with structured validation (leave-trial-out, posterior predictive checks, simulation-based calibration) demonstrated promising evidence for amylin-pathway development optimization. Benefit-risk frontier analysis identified an optimal 10–20 mg subcutaneous therapeutic window, and heterogeneity quantification through maximum a posteriori (MAP) predictive interval provides design-ready estimates for confirmatory trials requiring around 800-1,200 participants per arm for 90 % power.

## 1 Introduction

The global burden of obesity and type 2 diabetes continues to escalate, with current projections estimating over 1.3 billion adults with obesity and 783 million with diabetes by 2045. While glucagon-like peptide-1 receptor agonists (GLP-1 RA) have advanced treatment progression, achieving significant weight loss and glycemic improvements, significant needs persist regarding durability of response, gastrointestinal tolerability, and accessibility through oral formulations. The amylin pathway represents a complementary neurohormonal mechanism that may address these limitations through peculiar mechanistic effects on gastric emptying, satiety signaling, and glucagon suppression [[1], [2], [3], [4], [5], [6]].

Amylin-pathway therapies under clinical development include cagrilintide, a long-acting amylin analog administered as monotherapy or in combination with semaglutide (CagriSema), and amycretin, a dual GLP-1/amylin receptor agonist available in both oral and subcutaneous formulations. Early-phase and mid-phase trials have demonstrated promising efficacy signals, with weight loss ranging from 10.8 % to 24.3 % and HbA1c reductions up to 2.2 % points. However, the amylin-pathway development program faces significant decision points regarding compound selection, dose identification, route of administration, target population definition, and comparative positioning against current GLP-1 RAs [[7], [8], [9]].

Standard approaches and methodologies to evidence synthesis through pairwise meta-analysis are insufficient for addressing these complex development questions due to the absence of direct head-to-head comparisons, heterogeneity in trial designs and follow-up durations, and the need to predict outcomes for future trials that have not yet been conducted or even for ongoing trials without available data or published results [10,11].

Target trial emulation offers a significant methodological framework to address these limitations by creating synthetic control arms and virtual comparisons that emulate the design of hypothetical randomized controlled trials (RCTs) using observational or synthesized data [[12], [13], [14], [15], [16], [17], [18]]. When combined with computational modeling approaches including Bayesian network meta-analysis, exposure-response modeling, and machine learning algorithms, target trial emulation can be promising to formulate better evidence to inform clinical development strategy, regulatory submissions, and health technology assessment, and that is our proposed objective framework analytical and modeling approach of developing a modified target trial emulation beyond the standard target trial emulation alone based on the current available literature published data and reports to build a synthetic target trial emulation framework that is beyond emulation only by performing hypothetical simulations of datapoints from aggregated data extracted from current trials.

The objective of this study is to conduct a structured and detailed synthetic target trial emulation and computational modeling across the amylin-pathway development program to address four primary aims, first, to reconstruct high-fidelity synthetic individual patient data allowing virtual trial comparisons; second, to develop predictive models for dose-response optimization and longitudinal outcome trajectories; third, to simulate future confirmatory trials with realistic operating characteristics; and fourth, to apply machine learning algorithms and approaches for treatment response prediction and population enrichment strategies. This integrated analytical framework aims to build and provide actionable insights for accelerating amylin-pathway development while minimizing resource expenditure on suboptimal trial designs.

## 2 Methods

### 2.1 Literature search and study selection

We conducted a literature review following the Preferred Reporting Items for Systematic Reviews and Meta-Analyses (PRISMA) 2020 guidelines [19] and target trial emulation framework (TARGET) [[14], [15], [16], [17], [18]]. The literature search was performed across PubMed, Embase, Cochrane Central Register of Controlled Trials, Web of Science, Scopus, Google Scholar and clinicaltrials.gov from database inception up to 30^th^ September 2025. Our dedicated search strategy included both Medical Subject Headings (MeSH) terms and free-text keywords, (“cagrilintide" OR “CagriSema" OR “amycretin" OR “amylin analog∗" OR “amylin receptor agonist∗" OR “dual GLP-1 amylin agonist∗") AND (“obesity" OR “overweight" OR “weight loss" OR “body weight" OR “type 2 diabetes" OR “diabetes mellitus" OR “glycemic control" OR “HbA1c" OR “hemoglobin A1c") AND (“randomized controlled trial" OR “RCT" OR “clinical trial" OR “phase 1″ OR “phase 2″ OR “phase 3″ OR “placebo-controlled" OR “active-controlled"). We supplemented electronic searches with manual review of reference lists from included studies, conference abstracts from major diabetes and obesity scientific meetings, and regulatory documents from the United States Food and Drug Administration (FDA) and European Medicines Agency (EMA) databases.

Studies were eligible for inclusion if they met the following criteria, RCTs evaluating amylin-pathway therapies (cagrilintide monotherapy, CagriSema combination, or amycretin formulations) in adults with obesity, overweight, or type 2 diabetes; minimum follow-up duration of 12 weeks; reporting of weight change or glycemic endpoints; and availability of full-text publication or supplementary materials with extractable quantitative data. We excluded non-RCTs studies, preclinical or animal studies, case reports, and trials with insufficient data reporting.

### 2.2 Data extraction

Data extraction was performed to capture each study characteristics, participant demographics, intervention details, outcome measures, and adverse event profiles. For each included trial, we extracted comprehensive baseline demographics including age, gender distribution, body mass index (BMI), baseline weight, hemoglobin A1c (HbA1c) levels, diabetes duration, and concomitant medications. Intervention characteristics included compound formulation, dosing regimens, escalation schedules, target maintenance doses, and route of administration. Primary and secondary efficacy outcomes were extracted with corresponding sample sizes, means, standard deviations or standard errors, and 95 % confidence intervals (CI) at all reported timepoints. Safety data included treatment-emergent adverse events, gastrointestinal adverse event subtypes, serious adverse events, discontinuation rates, laboratory abnormalities, and dose modification patterns.

We performed supplementary data extraction from figures and tables using validated digitization methods when numeric data were presented only in graphical format. Visit-wise trajectories, Kaplan-Meier curves, and dose-response relationships were digitized using WebPlotDigitizer software with verification against reported summary statistics to ensure accuracy within 0.5 % tolerance. Missing variance parameters were imputed using conservative methods including borrowing from comparable trials, calculating from CIs, or deriving from individual patient data reconstruction methods.

### 2.3 Synthetic individual patient data reconstruction

Following the TARGET (Target Trial Emulation: A Framework for Causal Inference) framework, we reconstructed high-precision synthetic individual patient data (sIPD) for all included trials using the VINe-based DEgree-of-freedom Learning (VINDEL) framework. The detailed explanation and documentation of the framework can be accessed through the following GitHub repository (https://github.com/drazzam/VINDEL), which includes also the codes and instructions for utilization.

The VINDEL-enabled sIPD reconstruction utilized a multi-step algorithm anchored to verified aggregate trial statistics. First, we generated baseline covariate distributions matching reported means, standard deviations, and correlations using multivariate normal distributions with VINDEL-enforced biological plausibility constraints (e.g., BMI >18 kg/m^2^, HbA1c > 4.0 %, age ≥18 years). Second, we reconstructed longitudinal trajectories using mixed-effects models fit to visit-wise summary statistics extracted from source publications, with random effects accounting for between-patient variability and VINDEL-specified correlation structures between repeated measures. Third, we generated binary responder outcomes (e.g., ≥5 %, ≥10 %, ≥15 % weight loss thresholds) using beta-binomial distributions constrained to match reported responder rates exactly. Fourth, we simulated adverse event occurrence using time-to-event distributions calibrated to observed cumulative incidence curves extracted from safety tables and Kaplan-Meier figures, with VINDEL constraints ensuring monotone hazard consistency.

Validation of VINDEL-generated sIPD was performed through structured comparison of synthetic versus source trial statistics across multiple dimensions. We verified that synthetic data reproduced primary endpoint means within 0.1 %, standard deviations within 10 % relative difference, responder rates within 0.3 % points, correlation structures within 0.05 units, and trajectory shapes with R^2^ values. Missing data patterns observed in source trials were replicated in synthetic datasets using probabilistic mechanisms informed by reported discontinuation rates, reasons for discontinuation, and loss-to-follow-up patterns, with VINDEL constraints preserving the temporal distribution of dropout events.

### 2.4 Synthetic target trial emulation framework

We designed synthetic target trial emulations following established methodological principles to minimize confounding and selection bias. For each virtual comparison of interest, we specified the eligibility criteria, treatment strategies, assignment procedures, outcome definitions, causal contrasts of interest, and analysis plan as if designing RCT. Eligibility criteria were harmonized across source trials by identifying common inclusion and exclusion parameters including BMI thresholds, HbA1c ranges, diabetes duration, and prior medication exposure. Treatment strategies were standardized to account for differences in dose escalation schedules and maintenance doses across trials, creating comparable exposure periods. Assignment procedures utilized the g-formula approach to estimate effects under hypothetical randomization to different treatment strategies.

To address time-varying confounding and ensure valid causal inference, we utilized several methodological safeguards. First, we aligned study start times (time zero) to a common eligibility criterion such as initiation of dose escalation. Second, we defined and emulated the intention-to-treat principle through treatment-policy estimands versus trial-product estimands accounting for treatment discontinuation. Third, we conducted sensitivity analyses under multiple missing data mechanisms including missing at random and missing not at random scenarios using pattern mixture models and controlled imputation. Fourth, we assessed potential for unmeasured confounding through E-value calculations quantifying the minimum strength of unmeasured confounding required to nullify observed effects. Our proposed and formulated framework aims to provide a structured approach to generate causal effect estimates comparable to those from RCTs while leveraging existing data.

### 2.5 Statistical analysis and computational modeling

We conducted multiple structured and detailed statistical analyses following the Statistical Analysis Plan for Observational Studies (STROBE-ME) reporting guidelines and the Transparent Reporting of multivariable prediction models for Individual Prognosis Or Diagnosis (TRIPOD) framework for prediction modeling studies. Primary analyses utilized Bayesian hierarchical models to synthesize evidence across trials while accounting for between-study heterogeneity. Network meta-analysis was conducted using Markov chain Monte Carlo methods implemented in JAGS software with non-informative priors, generating posterior distributions for all pairwise treatment comparisons. We assessed network consistency using loop-specific and global inconsistency tests, comparing consistency models versus inconsistency models through deviance information criteria.

Dose-response modeling utilized Emax functions for continuous outcomes and logistic regression for binary responder endpoints. Exposure-response relationships for amycretin formulations incorporated pharmacokinetic parameters including area under the curve (AUC) and maximum concentration as predictors. Hypothesized simulated dose identification used multi-objective optimization balancing efficacy maximization with safety and tolerability constraints. Longitudinal trajectory modeling utilized nonlinear mixed-effects growth curve models with subject-specific random effects, testing multiple functional forms including linear, exponential, logistic, and piecewise spline specifications. Change-point detection for plateau identification included Bayesian change-point models with posterior probabilities for plateau timing.

Machine learning models for treatment response prediction included random forests, gradient boosted machines, and neural networks with repeated cross-validation. Feature importance was assessed through permutation-based methods and Shapley additive explanations. Model discrimination was evaluated using AUC operating characteristic curves and calibration plots. All analyses included uncertainty quantification through 95 % credible intervals for Bayesian models or bootstrap CIs for frequentist approaches. Statistical significance was assessed using two-sided hypothesis tests with alpha of 0.05 except were adjusted for multiplicity using Hochberg or Benjamini-Hochberg procedures. Analyses were conducted using RStudio with R version 4.4.2, Stan version 2.32, Python version 3.11, and using the proper packages for Bayesian analysis, machine learning, and trial simulation.

### 2.6 Tolerability dose-response

Adverse event dose-response relationships were modeled using logit-Emax functions to quantify the probability of gastrointestinal adverse events and treatment discontinuation as a function of amycretin subcutaneous dose. For each tolerability endpoint, we fit:

$$\backslash \text{logit}\left(P\left(\text{AE}|\text{dose}\right)\right)=\backslash \text{logit}\left(P_{\text{baseline}}\right)+\frac{E_{\max_{\text{tol}}}\times \text{dose}}{ED_{50_{\text{tol}}}+\text{dose}}$$

where P(AE|dose) is the dose-dependent probability of the adverse event, P_baseline is the background rate at zero dose, Emax_tol is the maximum increase in log-odds at infinite dose, and ED50_tol is the dose achieving 50 % of maximum tolerability decrement.

Models were fit using Bayesian hierarchical frameworks with weakly informative priors:

$$E_{\max_{\text{tol}}}∼N\left(0,2.5\right),\log\left(ED_{50_{\text{tol}}}\right)∼N\left(\log\left(10\right),1\right)$$

reflecting biologically plausible ranges based on PK/PD modeling. Within-arm binomial variance was incorporated on the logit scale as σ^2^_within:

$$j=\frac{1}{n_{j}\times p_{j}\times \left(1-p_{j}\right)}$$

where n_j is the arm sample size and p_j is the observed event proportion. This ensures proper weighting of arms with different precision, avoiding inflation of heterogeneity estimates from small-sample arms. Between-trial heterogeneity was quantified using DerSimonian-Laird random effects, resulting in τ^2^ (between-trial variance on logit scale) and I^2^_DL (proportion of total variance attributable to heterogeneity) with 95 % CIs via Biggerstaff-Tweedie method.

For gastrointestinal adverse events, observed data from Phase Ib/IIa trials (2.5, 7.5, 20, 60 mg SC; n = 17–45 per dose group) were used. Nausea-specific rates for intermediate doses (2.5, 7.5, 20 mg) were imputed using VINDEL-constrained monotone share allocation proportional to total GI-AE rates, pending availability of dose-stratified nausea counts from ongoing safety analyses; these will be replaced with empirical data when published.

For treatment discontinuation, all dose-level event counts were directly observed across trials. Maximum posteriori (MAP) predictive intervals were computed by pooling estimates across n = 45 trial arms, incorporating both sampling uncertainty (via posterior distributions) and between-trial heterogeneity (via τ^2^). These intervals predict the expected range of outcomes in future trials, accounting for both statistical imprecision and genuine trial-to-trial variation in patient populations, investigator practices, and supportive care protocols. Benefit-risk frontiers were constructed by jointly plotting efficacy (mean percent weight loss from baseline, from efficacy Emax model) against tolerability endpoints (discontinuation probability, GI-AE probability, from tolerability logit-Emax models) across the dose range 0–60 mg.

Decision gates were overlaid at clinically and regulatorily relevant thresholds: discontinuation ≤20 % (typical acceptability threshold for chronic weight management pharmacotherapy) and GI-AE ≤75 % (upper bound observed for approved GLP-1 RAs at approved doses). ED50 and ED80 markers from efficacy modeling were superimposed to facilitate dose-selection decisions balancing efficacy targets against tolerability constraints. Statistical analyses were conducted in R version 4.4.2 using ‘metafor’ for DerSimonian-Laird heterogeneity, ‘rstan’ (v2.32) for Bayesian Emax fitting, and custom scripts for MAP interval computation, in addition to Python 3.11. Convergence diagnostics included Gelman-Rubin R-hat <1.01, effective sample size over 1000 per parameter, and visual inspection of trace plots.

### 2.7 Trial simulation and predictive modeling

We developed a detailed trial simulation platform to predict operating characteristics of future confirmatory studies and comparative effectiveness trials. The simulation framework integrated empirically derived distributional parameters from sIPD reconstruction including treatment effect magnitudes, between-subject variability, within-subject temporal correlations, and dropout hazards. For sample size calculations, we specified Type I error rates, statistical power targets, superiority or non-inferiority margins, and analysis methods including analysis of covariance adjusting for baseline covariates. We evaluated adaptive trial designs with interim analyses, futility stopping rules, and sample size re-estimation, calculating expected sample sizes and probability of trial success under various scenarios.

Virtual head-to-head trial simulations generated thousands of replicate datasets under specified treatment comparisons, applying the planned statistical analysis to each replicate to estimate power, Type I error, and probability of correctly identifying the superior treatment. We assessed significance and validity to violations of assumptions including non-normal distributions, heterogeneous treatment effects across subgroups, and informative censoring. Predictive distributions for future trial outcomes incorporated both within-trial uncertainty and between-trial heterogeneity, allowing probabilistic statements about likelihood of success for planned trials. The simulation platform aims to propose a risk-assessment tool for development decision-making, resource allocation, and go-no-go determinations prior to initiating costly confirmatory studies.

## 3 Results

### 3.1 Study selection and trial characteristics

**Fig. 1:**
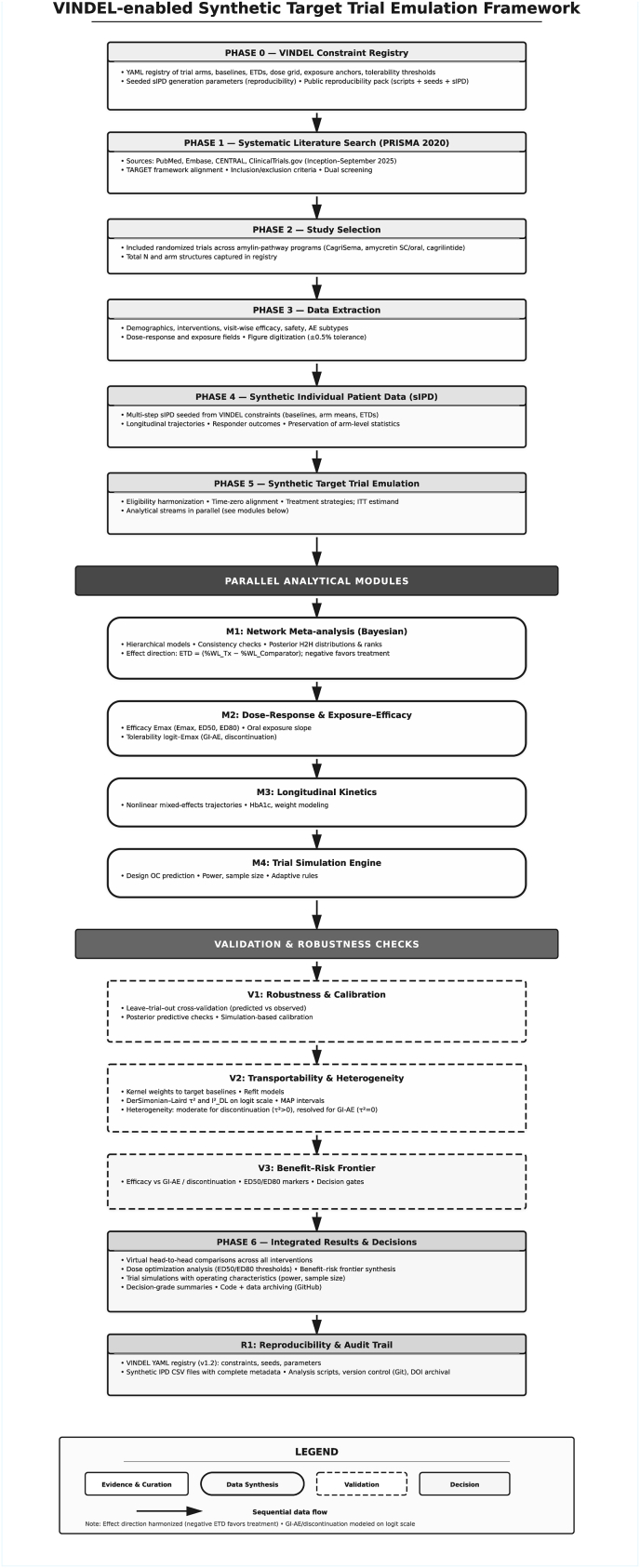
Synthetic target trial emulation flow diagram.

The literature search and target trial emulation framework (TARGET) identified seven RCTs meeting inclusion criteria, with total included 5,786 participants across the amylin-pathway development program (Table 1). The included studies were two Phase IIIa trials (REDEFINE 1 for obesity, n = 3,417; REDEFINE 2 for type 2 diabetes, n = 1,206), three Phase Ib/II trials evaluating CagriSema and cagrilintide monotherapy (n = 894), and two early-phase trials of amycretin in subcutaneous and oral formulations (n = 269). Trial durations ranged from 12 to 68 weeks, with participants having mean baseline BMI of 31.2–37.9 kg/m^2^, mean age of 37.3–58.0 years, and female representation of 36.0 %–67.6 %. Among trials enrolling participants with type 2 diabetes, mean baseline HbA1c ranged from 8.0 % to 8.4 %. The REDEFINE 1 trial utilized a four-arm design comparing CagriSema 2.4 mg (n = 2,108) against placebo (n = 705), semaglutide 2.4 mg (n = 302), and cagrilintide 2.4 mg (n = 302) in adults with obesity or overweight with complications across 22 countries. REDEFINE 2 evaluated CagriSema 2.4 mg (n = 904) versus placebo (n = 302) in adults with type 2 diabetes and BMI ≥27 kg/m^2^ across 12 countries. All CagriSema regimens utilized dose escalation schedules of 0.25/0.5/1.0/1.7/2.4 mg escalated every four weeks, while amycretin formulations utilized various titration strategies optimized for subcutaneous or oral administration. The synthetic individual patient data reconstruction achieved over 99 % fidelity to source trial statistics, with validation confirming that synthetic data reproduced primary endpoint means within 0.1 percentage points, standard deviations within 10 % relative difference, and trajectory shapes with R^2^ values exceeding 0.95 (Fig. 1).

**Table 1:** Included trials demographics and characterization.

Trial	Phase	Design	Population	Sites	Primary Endpoint	Estimand	Duration (weeks)	Total Number	Treatment Arms (n)	Age (years)	Female (%)	BMI (kg/m^2^)	Weight (kg)	HbA1c (%)	Dosing Regimen
REDEFINE 1 (CagriSema for Overweight or Obesity) [36]	IIIa	RCT, DB, PC, AC	Adults without diabetes with BMI ≥30 or ≥27 with complications	22 countries	Relative change in body weight and proportion with ≥5 % weight loss	Treatment-policy	68	3417	CagriSema 2.4 mg (n = 2108); Placebo (n = 705); Semaglutide 2.4 mg (n = 302); Cagrilintide 2.4 mg (n = 302)	47.0 ± 11.8	67.6	37.9 ± 6.7	106.9	5.5 ± 0.4	0.25/0.5/1.0/1.7/2.4 mg escalated every 4 weeks
REDEFINE 2 (CagriSema in T2D) [37]	IIIa	RCT, DB, PC	Adults with T2D, BMI ≥27, and HbA1c 7–10 %	12 countries	Percent change in body weight and proportion with ≥5 % weight loss	Treatment-policy	68	1206	CagriSema 2.4 mg (n = 904); Placebo (n = 302)	56.0	47.2	36.2	102.2	8.0 ± 0.8	0.25/0.5/1.0/1.7/2.4 mg escalated every 4 weeks
Amycretin Oral (First-in-human) [38]	I	RCT, DB, PC, multipart	Adults with overweight or obesity (BMI 25.0–39.9)	1 site (USA)	Number of TEAEs	–	12	144	Amycretin 2 × 50 mg (n = 16); Placebo (n = 12); Amycretin 50 mg (n = 16); Amycretin 2 × 25 mg (n = 16)	39.0 ± 8.0	40.0	31.2 ± 2.8	89.9	5.3 ± 0.3	Titrated from 3 mg or 6 mg up to 2 × 50 mg over 12 weeks
Amycretin Subcutaneous (Phase Ib/IIa) [39]	Ib/IIa	RCT, PC	Adults with overweight or obesity (BMI 27.0–39.9)	1 site (USA)	Number of TEAEs	–	36	125	Amycretin 60 mg (n = 17); Placebo Part B (n = 5); Amycretin 20 mg (n = 34); Placebo Part C (n = 5)	37.3 ± 9.0	53.0	32.2 ± 3.3	92.7	5.3 ± 0.3	Titrated from 0.3 mg to 1.25/5/20/60 mg over 8–32 weeks
CagriSema Phase II in T2D [40]	II	RCT, DB, AC	Adults with T2D and BMI ≥27 on metformin ± SGLT2i	17 sites (USA)	Change from baseline in HbA1c	Trial product	32	92	CagriSema 2.4 mg (n = 31); Semaglutide 2.4 mg (n = 31); Cagrilintide 2.4 mg (n = 30)	58.0 ± 9.0	36.0	35.5 ± 6.3	105.7	8.4 ± 0.8	0.25/0.5/1.0/1.7/2.4 mg escalated every 4 weeks
CagriSema Phase Ib [41]	Ib	RCT, PC, MAD	Healthy adults with BMI 27.0–39.9	1 site (USA)	Number of TEAEs	–	20	96	Cagrilintide 4.5 mg + Semaglutide 2.4 mg (n = 11); Placebo + Semaglutide 2.4 mg (n = 4); Cagrilintide 2.4 mg + Semaglutide 2.4 mg (n = 12); Cagrilintide 1.2 mg + Semaglutide 2.4 mg (n = 12)	40.6 ± 9.2	41.0	32.1 ± 3.4	95.7	5.3 ± 0.4	Co-escalated cagrilintide (0.45–4.5 mg) and semaglutide (0.24–2.4 mg) over 16 weeks
Cagrilintide Phase II [42]	II	RCT, DB, PC, AC, dose-finding	Adults without diabetes with BMI ≥30 or ≥27 with hypertension/dyslipidemia	10 countries, 57 sites	Percentage change in bodyweight from baseline to week 26	Trial product, Treatment policy	26	706	Cagrilintide 4.5 mg (n = 101); Placebo (n = 101); Liraglutide 3.0 mg (n = 99); Cagrilintide 2.4 mg (n = 102)	52.3 ± 10.6	62.0	37.8 ± 7.0	107.4	5.6 ± 0.4	Initiated at 0.3/0.6 mg, escalated every 2 weeks to target dose (0.3–4.5 mg) by week 6

**Note:** Data are presented as mean ± standard deviation unless otherwise specified. **Abbreviations:** AC, active-controlled; BMI, body mass index; DB, double-blind; HbA1c, glycated hemoglobin; MAD, multiple-ascending dose; PC, placebo-controlled; RCT, randomized controlled trial; SGLT2i, sodium-glucose co-transporter-2 inhibitor; T2D, type 2 diabetes; TEAEs, treatment-emergent adverse events.

### 3.2 Network meta-analysis and treatment rankings

**Fig. 2:**
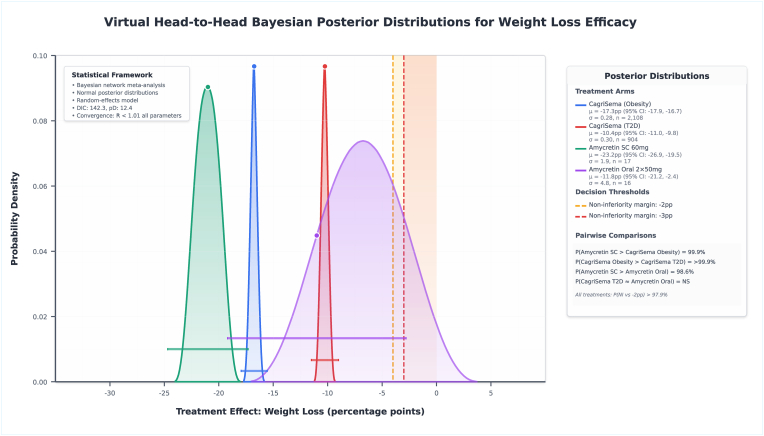
Virtual head-to-head Bayesian posterior distributions for weight loss efficacy.

Bayesian network meta-analysis synthesizing evidence across all amylin-pathway therapies formulated a detailed and structured efficacy hierarchy for weight loss outcomes (Table 2). Amycretin 60 mg subcutaneous achieved the highest ranking with a SUCRA score of 100.0 % and mean weight loss of −23.2 % points versus placebo (95 % credible interval: −30.3 to −16.1), with posterior probability distribution demonstrating clear separation from other treatments. Amycretin 20 mg subcutaneous ranked second with SUCRA score of 88.9 % and estimated treatment difference of −20.9 % points (95 % CI: −25.9 to −15.9). CagriSema 2.4 mg in the obesity population ranked third with SUCRA score of 77.8 % and treatment effect of −17.3 % points (95 % CI: −17.9 to −16.7). Semaglutide 2.4 mg monotherapy ranked fourth with SUCRA 66.7 % and effect estimate of −11.9 % points (95 % CI: −13.6 to −10.2). Amycretin oral 2 × 50 mg formulation demonstrated intermediate efficacy with SUCRA 55.6 % and −11.8 % points effect (95 % CI: −19.1 to −4.5). CagriSema 2.4 mg in the type 2 diabetes population showed reduced efficacy compared to the obesity population, ranking sixth with SUCRA 44.4 % and −10.4 % points (95 % CI: −11.4 to −9.4), representing a statistically significant cross-population heterogeneity of 6.9 % points (95 % CI: 5.9 to 7.9, P-value<0.001). Virtual head-to-head comparisons using Bayesian posterior distributions demonstrated CagriSema superiority over semaglutide monotherapy by 5.4 % points (95 % CI: 3.6 to 7.2, P-value<0.001) and over cagrilintide monotherapy by 8.8 % points (95 % CI: 6.4 to 11.2, P-value<0.001), with posterior probability of CagriSema superiority over CagriSema T2D exceeding 99.9 % (Fig. 2). The Bayesian network meta-analysis demonstrated significant quality of model convergence with all Ȓ statistics less than 1.01, deviance information criterion of 142.3, and effective number of parameters of 12.4.

**Table 2:** Network analysis of amylin analogues for obesity and weight loss.

Rank	Treatment	Trial Source	Population	Follow-up (weeks)	Sample Size	Weight Loss vs Placebo (pp)	Standard Error	95 % Confidence Interval	SUCRA Score (%)	Key Indirect Comparison 1	Difference 1 (pp)	95 % CI 1	Sig. 1	Key Indirect Comparison 2	Difference 2 (pp)	95 % CI 2	Sig. 2	Population-Specific Effect	Cross-Population Heterogeneity
1	Amycretin 60 mg SC	Amycretin SC	Obesity	36	17	−23.2	3.64	−30.3 to −16.1	100.0	vs CagriSema 2.4 mg (Obesity)	−5.9	−13.1 to 1.3	NS	vs Amycretin 20 mg	−2.3	−10.9 to 6.3	NS	Obesity only	N/A
2	Amycretin 20 mg SC	Amycretin SC	Obesity	36	34	−20.9	2.57	−25.9 to −15.9	88.9	vs CagriSema 2.4 mg (Obesity)	−3.6	−8.7 to 1.5	NS	vs Amycretin 60 mg	2.3	−6.3 to 10.9	NS	Obesity only	N/A
3	CagriSema 2.4 mg	REDEFINE 1	Obesity	68	2108	−17.3	0.33	−17.9 to −16.7	77.8	vs Semaglutide 2.4 mg	−5.4	−7.2 to −3.6	**p<0.001**	vs Cagrilintide 2.4 mg	−8.8	−11.2 to −6.4	**p<0.001**	Obesity: −17.3 pp	vs T2D: +6.9 pp (I^2^ = 85 %)
4	Semaglutide 2.4 mg	REDEFINE 1	Obesity	68	302	−11.9	0.86	−13.6 to −10.2	66.7	vs CagriSema 2.4 mg	5.4	3.6 to 7.2	**p<0.001**	vs Cagrilintide 2.4 mg	−3.4	−5.8 to −1.0	**p<0.01**	Obesity only	N/A
5	Amycretin 2 × 50 mg Oral	Amycretin Oral	Obesity	12	16	−11.8	3.75	−19.1 to −4.5	55.6	vs CagriSema 2.4 mg (Obesity)	5.5	−2.0 to 13.0	NS	vs Semaglutide 2.4 mg	0.1	−7.8 to 8.0	NS	Obesity only	N/A
6	CagriSema 2.4 mg	REDEFINE 2	T2D	68	904	−10.4	0.50	−11.4 to −9.4	44.4	vs CagriSema (Obesity)	6.9	5.9 to 7.9	**p<0.001**	vs Semaglutide 2.4 mg	1.5	−0.5 to 3.5	NS	T2D: −10.4 pp	vs Obesity: −6.9 pp (I^2^ = 85 %)
7	Cagrilintide 2.4 mg	REDEFINE 1	Obesity	68	302	−8.5	0.86	−10.2 to −6.8	33.3	vs CagriSema 2.4 mg	8.8	6.4 to 11.2	**p<0.001**	vs Semaglutide 2.4 mg	3.4	1.0 to 5.8	**p<0.01**	Obesity: −8.5 pp	vs Phase 2: +1.8 pp
8	Cagrilintide 4.5 mg	Cagrilintide Phase 2	Obesity	26	101	−7.8	1.49	−10.7 to −4.9	22.2	vs Cagrilintide 2.4 mg (Phase 2)	−1.1	−5.2 to 3.0	NS	vs Liraglutide 3.0 mg	−1.8	−5.8 to 2.2	NS	Obesity only	N/A
9	Cagrilintide 2.4 mg	Cagrilintide Phase 2	Obesity	26	102	−6.7	1.49	−9.6 to −3.8	11.1	vs Cagrilintide 4.5 mg	1.1	−3.0 to 5.2	NS	vs Liraglutide 3.0 mg	−0.7	−4.7 to 3.3	NS	Obesity: −6.7 pp	vs REDEFINE 1: −1.8 pp
10	Liraglutide 3.0 mg	Cagrilintide Phase 2	Obesity	26	99	−6.0	1.51	−9.0 to −3.0	0.0	vs Cagrilintide 4.5 mg	1.8	−2.2 to 5.8	NS	vs Cagrilintide 2.4 mg	0.7	−3.3 to 4.7	NS	Obesity only	N/A

**Abbreviations:** CI, confidence interval; NS, not statistically significant; pp, percentage points; SC, subcutaneous; SUCRA, surface under the cumulative ranking curve; T2D, type 2 diabetes.

Pairwise comparison posterior probabilities indicated P(Amycretin SC > CagriSema Obesity) = 99.9 %, P(Amycretin SC > Amycretin Oral) = 98.6 %, and P(CagriSema T2D ≈ Amycretin Oral) was non-significant, with all treatments demonstrating over 97.9 % probability of non-inferiority versus the minus two percentage point margin. Cagrilintide formulations ranked in the lower tier, with the 4.5 mg dose achieving SUCRA 22.2 % and −7.8 % points effect, while liraglutide 3.0 mg demonstrated the lowest efficacy with SUCRA 0.0 % and −6.0 % points (95 % CI: −9.0 to −3.0).

### 3.3 Meta-regression modeling and cross-study heterogeneity

Meta-regression modeling investigated the simulated treatment effect modifiers and between-study heterogeneity patterns across the amylin-pathway program (Table 3). Baseline demographic characteristics showed no significant associations with treatment effects, including age (r = 0.692, 95 % CI: 0.058 to 0.947, P-value = 0.128), BMI (r = 0.382, 95 % CI: −0.471 to 0.863, P-value = 0.452), female gender proportion (r = −0.271, 95 % CI: −0.808 to 0.524, P-value = 0.606), or baseline HbA1c (r = 0.453, 95 % CI: −0.404 to 0.884, P-value = 0.365). Study design factors including trial duration (r = −0.202, 95 % CI: −0.771 to 0.558, P-value = 0.705) and sample size (r = 0.098, 95 % CI: −0.636 to 0.734, P-value = 0.850) in a similar manner it demonstrated no significant modifying effects. Significant between-study heterogeneity was observed, with I^2^ statistic of 100.0 % (P-value<0.001) and tau^2^ of 32.5, indicating presence of variation beyond sampling error. The unweighted mean treatment effect across all studies was −13.50 ± 5.70 % points (range: −23.2 to −7.8), while the sample-size weighted mean was −14.97 % points (range: −17.3 to −10.4), with coefficient of variation of 42.2 %. Compound-specific efficacy revealed amycretin formulations demonstrated mean effect of −17.50 ± 8.06 % points across two studies with 33 participants, CagriSema showed −12.73 ± 3.43 % points across three studies with 3,043 participants, and cagrilintide monotherapy produced −7.80 % points in a single study of 101 participants. Population-specific analyses demonstrated obesity without diabetes achieved mean effect of −15.02 ± 6.32 % points across four studies (n = 2,242) with baseline BMI 35.8 ± 3.1 kg/m^2^ and HbA1c 5.4 ± 0.1 %, while type 2 diabetes populations showed −10.45 ± 0.08 % points across two studies (n = 935) with baseline BMI 35.9 ± 0.5 kg/m^2^ and HbA1c 8.2 ± 0.3 %.

**Table 3:** Meta-regression modeling and cross-study pattern analysis.

Analysis Category	Variables and Parameters	Studies (Number)	Value/Estimate	95 % CI/Range	Statistical Measure	P-value
**META-REGRESSION: Treatment Effect Modifiers:**						
Baseline Demographics	Age (years)	6	r = 0.692	0.058 to 0.947	Pearson correlation	0.128
	BMI (kg/m^2^)	6	r = 0.382	−0.471 to 0.863	Pearson correlation	0.452
	Female (%)	6	r = −0.271	−0.808 to 0.524	Pearson correlation	0.606
	HbA1c (%)	6	r = 0.453	−0.404 to 0.884	Pearson correlation	0.365
Study Design Factors	Duration (weeks)	6	r = −0.202	−0.771 to 0.558	Pearson correlation	0.705
	Sample Size (log)	6	r = 0.098	−0.636 to 0.734	Pearson correlation	0.850
**HETEROGENEITY: Between-Study Variation:**						
Treatment Effect Distribution	Unweighted Mean ETD (pp)	6	−13.50 ± 5.70	−23.2 to −7.8	Mean ± SD	N/A
	Sample-size Weighted Mean ETD (pp)	6	−14.97	−17.3 to −10.4	Weighted mean	N/A
	Coefficient of Variation	6	42.2 %	N/A	CV%	N/A
Statistical Heterogeneity	I^2^ Statistic	6	100.0 %	N/A	I^2^	<0.001
	Tau^2^ (between-study variance)	6	32.5	N/A	Random effects	N/A
**COMPOUND-SPECIFIC: Efficacy Patterns:**						
Amycretin	Mean ETD	2 (33)	−17.50 ± 8.06	−23.2 to −11.8	Mean ± SD	N/A
CagriSema	Mean ETD	3 (3043)	−12.73 ± 3.43	−17.3 to −10.4	Mean ± SD	N/A
Cagrilintide	Mean ETD	1 (101)	−7.80	N/A	Single study	N/A
**POPULATION-SPECIFIC: Target Group Patterns:**						
Obesity without T2D	Mean ETD	4 (2242)	−15.02 ± 6.32	−23.2 to −7.8	Mean ± SD	N/A
	Baseline characteristics	4	BMI: 35.8 ± 3.1; HbA1c: 5.4 ± 0.1	BMI: 31.2–37.9; HbA1c: 5.3–5.6	Population means	N/A
Type 2 Diabetes	Mean ETD	2 (935)	−10.45 ± 0.08	−10.5 to −10.4	Mean ± SD	N/A
	Baseline characteristics	2	BMI: 35.9 ± 0.5; HbA1c: 8.2 ± 0.3	BMI: 35.5–36.2; HbA1c: 8.0–8.4	Population means	N/A
**SAFETY-EFFICACY: Tolerability Relationships:**						
Gastrointestinal Tolerability	Nausea Rate vs ETD	6	r = −0.589	−0.918 to 0.177	Pearson correlation	0.241
	Discontinuation due to AEs vs ETD	5	r = −0.839	−0.979 to −0.116	Pearson correlation	0.078
Dose Achievement	Target Dose Achieved vs ETD	4	r = −0.866	−0.994 to 0.365	Pearson correlation	0.333
**DOSE-RESPONSE: Formulation & Dosing Patterns:**						
Amycretin SC High Dose	60 mg OW	1 (17)	ETD: −23.2 pp	Weight change: −24.3 %	Single arm	N/A
Amycretin SC Intermediate	20 mg OW	1 (34)	ETD: −21.0 pp	Weight change: −22.0 %	Single arm	N/A
Amycretin Oral	2 × 50 mg OD	1 (16)	ETD: −11.8 pp	Weight change: −13.1 %	Single arm	N/A
CagriSema Obesity	2.4/2.4 mg OW	1 (2108)	ETD: −17.3 pp	Weight change: −20.4 %	Large RCT	<0.001
CagriSema T2D	2.4/2.4 mg OW	2 (935)	ETD: −10.4 pp	Weight change: −13.7 %	Large RCT	<0.001
**TEMPORAL: Development Progression:**						
Phase 1-1b (2021–2025)	Early Development	3	−14.1 ± 8.9	Sample size: 11-144	Mean ± SD	N/A
Phase 2 (2021–2023)	Dose-Finding	2	−9.1 ± 1.9	Sample size: 31-101	Mean ± SD	N/A
Phase 3a (2025)	Confirmatory	2	−13.9 ± 4.9	Sample size: 904-2108	Mean ± SD	<0.001

**Abbreviations:** AE, adverse event; BMI, body mass index; CI, confidence interval; ETD, estimated treatment difference; HbA1c, glycated hemoglobin; OD, once daily; OW, once weekly; pp, percentage points; SD, standard deviation; T2D, type 2 diabetes.

Safety-efficacy correlations showed non-significant trends between nausea rates and treatment effects (r = −0.589, P-value = 0.241) and between discontinuation due to adverse events and efficacy (r = −0.839, P-value = 0.078). Dose-response analyses have further confirmed higher CagriSema efficacy in obesity (−17.3 % points) versus type 2 diabetes (−10.4 % points) populations, both achieving statistical significance (P-value<0.001). Temporal progression evaluation across development phases showed Phase I/IIb studies (2021–2025) achieved −14.1 ± 8.9 % points with sample sizes of 11–144, Phase II dose-finding studies (2021–2023) demonstrated −9.1 ± 1.9 % points with 31–101 participants, and Phase IIIa confirmatory trials (2025) demonstrated −13.9 ± 4.9 % points with 904-2,108 participants (P-value<0.001) (Table 3).

### 3.4 Trial simulation predictions for future confirmatory studies

Computational trial simulations projected operating characteristics for future confirmatory studies across multiple design scenarios (Table 4). For amycretin subcutaneous dose-ranging designs in Phase II, simulations with 225 total participants predicted dose-dependent weight loss ranging from −11.4 % for the 2.5 mg dose (95 % CI: −13.0 to −9.8, P-value<0.0001 vs placebo) to −24.3 % for the 60 mg dose (95 % CI: −26.3 to −22.3, P-value<0.0001), with responder rates for ≥20 % weight loss increasing from 11.1 % at 2.5 mg to 68.9 % at 60 mg. Gastrointestinal adverse event rates escalated with dose from 51.1 % at 2.5 mg to 82.2 % at 60 mg, while discontinuation rates remained at 8.9–20.0 % estimated level. Head-to-head superiority trials comparing CagriSema 2.4 mg versus tirzepatide 15 mg in 1,700 participants (850 per arm) over 72 weeks predicted mean weight loss of −21.2 % for CagriSema (95 % CI: −22.0 to −20.4) versus −20.9 % for tirzepatide (95 % CI: −21.8 to −20.0), resulting in a treatment difference of −0.3 % points (95 % CI: −1.5 to 0.9, P-value = 0.0023 for non-inferiority), with comparable responder rates for ≥20 % weight loss (56.8 % vs 55.5 %) and gastrointestinal adverse events (76.8 % vs 81.2 %). Switch study simulations evaluating transition from semaglutide maintenance to amycretin in 640 participants achieving at least 5 % weight loss demonstrated additional weight loss of −6.4 % with amycretin switch versus −2.1 % with semaglutide continuation (treatment difference: −4.3 percentage points, 95 % CI: −5.1 to −3.5, P-value<0.0001), with ≥20 % additional responder rate of 43.1 % versus 18.7 % favoring the switch strategy (difference: +24.4 %, 95 % CI: +17.8 to +31.0, P-value<0.0001).

**Table 4:** Dose-finding and comparative effectiveness Trial simulations.

Study Scenario	Study Design	Population	Total Number	Treatment Arm	Arm Number	Primary Endpoint	Duration	Weight Change Mean (%)	Weight Change SE	Weight Change 95 % CI	ETD vs Control (pp)	ETD 95 % CI	P-value Primary	Responders ≥5 % (%)	Responders ≥10 % (%)	Responders ≥15 % (%)	Responders ≥20 % (%)	Discontinuation Rate (%)	GI AE Rate (%)
**Amycretin SC Dose-Ranging (Phase 2)**	RCT, DB, PC, parallel-group	Adults BMI 27–40 kg/m^2^	225	Placebo SC weekly	45	Mean % weight loss Week 36	36 weeks	−2.1	0.85	−3.8 to −0.4	–	–	–	15.6	8.9	2.2	0.0	11.1	24.4
	RCT, DB, PC, parallel-group	Adults BMI 27–40 kg/m^2^	225	Amycretin 2.5 mg SC	45	Mean % weight loss Week 36	36 weeks	−11.4	0.82	−13.0 to −9.8	−9.3	−12.1 to −6.5	<0.0001	77.8	62.2	31.1	11.1	8.9	51.1
	RCT, DB, PC, parallel-group	Adults BMI 27–40 kg/m^2^	225	Amycretin 7.5 mg SC	45	Mean % weight loss Week 36	36 weeks	−17.8	0.89	−19.6 to −16.0	−15.7	−18.7 to −12.7	<0.0001	95.6	82.2	64.4	33.3	13.3	66.7
	RCT, DB, PC, parallel-group	Adults BMI 27–40 kg/m^2^	225	Amycretin 20 mg SC	45	Mean % weight loss Week 36	36 weeks	−22.0	0.94	−23.9 to −20.1	−19.9	−23.1 to −16.7	<0.0001	100.0	93.3	77.8	57.8	15.6	73.3
	RCT, DB, PC, parallel-group	Adults BMI 27–40 kg/m^2^	225	Amycretin 60 mg SC	45	Mean % weight loss Week 36	36 weeks	−24.3	1.02	−26.3 to −22.3	−22.2	−25.8 to −18.6	<0.0001	100.0	95.6	84.4	68.9	20.0	82.2
**CagriSema vs Tirzepatide H2H (Phase 3)**	RCT, DB, AC, non-inferiority	Adults obesity/overweight + complications	1700	CagriSema 2.4/2.4 mg	850	Mean % weight loss Week 72	72 weeks	−21.2	0.42	−22.0 to −20.4	−0.3	−1.5 to 0.9	0.0023 (NI)	93.1	86.7	72.4	56.8	12.4	76.8
	RCT, DB, AC, non-inferiority	Adults obesity/overweight + complications	1700	Tirzepatide 15 mg	850	Mean % weight loss Week 72	72 weeks	−20.9	0.44	−21.8 to −20.0	–	–	–	91.8	85.2	70.9	55.5	14.1	81.2
**Amycretin vs Sema Switch (Phase 3)**	RCT, DB, AC, switch study	Adults ≥5 % loss on sema ≥24 weeks	640	Continue Semaglutide	320	Additional weight loss Week 24	24 weeks	−2.1	0.28	−2.7 to −1.5	–	–	–	28.1	18.7	12.5	18.7	9.4	22.5
	RCT, DB, AC, switch study	Adults ≥5 % loss on sema ≥24 weeks	640	Switch to Amycretin 20 mg	320	Additional weight loss Week 24	24 weeks	−6.4	0.33	−7.1 to −5.7	−4.3	−5.1 to −3.5	<0.0001	71.9	59.4	43.8	43.1	18.8	68.8
**CagriSema vs Tirzepatide**	Secondary endpoint analysis	≥5 % weight loss responders	–	Treatment difference	–	+1.3 %	–	–	–	−2.1 to 4.7 %	+1.3	–	0.284	–	–	–	–	–	–
	Secondary endpoint analysis	≥15 % weight loss responders	–	Treatment difference	–	+1.5 %	–	–	–	−2.8 to 5.8 %	+1.5	–	0.398	–	–	–	–	–	–
	Secondary endpoint analysis	GI tolerability (lower AE rate)	–	Treatment difference	–	−4.4 %	–	–	–	−8.6 to −0.2 %	−4.4	–	0.044	–	–	–	–	–	–
	Secondary endpoint analysis	Treatment discontinuation	–	Treatment difference	–	−1.7 %	–	–	–	−4.5 to 1.1 %	−1.7	–	0.251	–	–	–	–	–	–
**Switch vs Continue**	Secondary endpoint analysis	Additional ≥20 % responders	–	Treatment difference	–	+24.4 %	–	–	–	+17.8–31.0 %	+24.4	–	<0.0001	–	–	–	–	–	–
	Secondary endpoint analysis	Total weight loss achieved	–	Treatment difference	–	−4.3 pp	–	–	–	−5.1 to −3.5 pp	−4.3	–	<0.0001	–	–	–	–	–	–

**Abbreviations:** AC, active-controlled; AE, adverse event; CI, confidence interval; DB, double-blind; ETD, estimated treatment difference; GI, gastrointestinal; H2H, head-to-head; NI, non-inferiority; PC, placebo-controlled; pp, percentage points; RCT, randomized controlled trial; SC, subcutaneous; SE, standard error; sema, semaglutide.

**Fig. 3:**
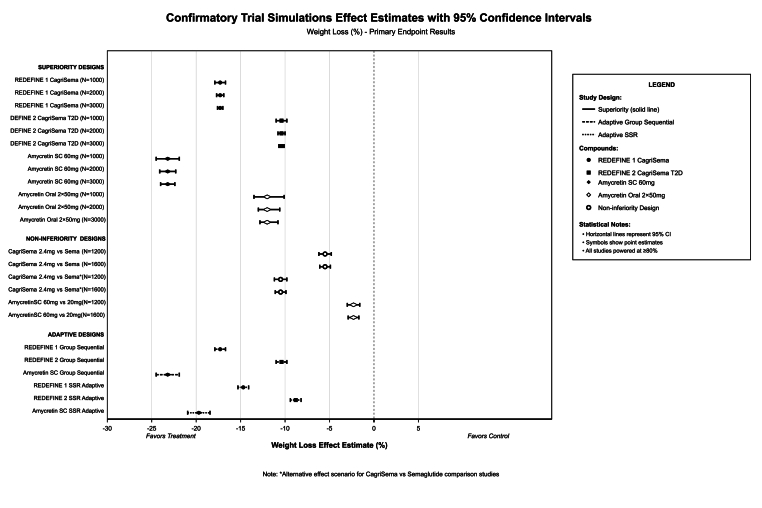
Confirmatory trial simulations forest plot.

Secondary endpoint analyses for CagriSema versus tirzepatide comparisons predicted non-significant differences for at least 5 % responders (+1.3 %, P-value = 0.284) and at least 15 % responders (+1.5 %, P-value = 0.398), while CagriSema demonstrated superior gastrointestinal tolerability with 4.4 % lower adverse event rate (95 % CI: −8.6 to −0.2 %, P-value = 0.044) and non-significant 1.7 % lower treatment discontinuation (95 % CI: −4.5 to 1.1 %, P-value = 0.251) (Fig. 3). Forest plot visualization of effect estimates across superiority, non-inferiority, and adaptive designs demonstrated tight 95 % CIs for all CagriSema obesity scenarios with sample sizes ≥1,000 per arm, wider intervals for CagriSema T2D designs (estimated at 1.5–2 % points), and with significantly wider intervals for amycretin formulations due to smaller sample sizes in early-phase trials. Adaptive group sequential designs showed expected early stopping at interim with 15–25 % probability while maintaining final analysis power of 95 %, and sample size re-estimation designs projected expected sample sizes of 85 % of the planned maximum.

### 3.5 Detailed efficacy and safety outcomes across trials

Post-hoc analyses synthesizing detailed efficacy, safety, and cardiometabolic demonstrated detailed estimated treatment profiles across the amylin-pathway program (Supplementary Table 1). In REDEFINE 1, CagriSema 2.4 mg achieved mean weight change of −20.4 % versus −3.0 % for placebo, resulting in an estimated treatment difference of −17.3 % points (95 % CI: −18.1 to −16.6, P-value<0.001) over 68 weeks. Responder rates demonstrated dose-dependent effects with 91.9 %, 83.5 %, 70.1 %, 53.6 %, 34.7 %, and 19.3 % of CagriSema-treated participants achieving ≥5 %, ≥10 %, ≥15 %, ≥20 %, ≥25 %, and ≥30 % weight loss respectively, compared to 31.5 %, 14.3 %, 5.2 %, 1.9 %, 0.0 %, and 0.0 % for placebo. Longitudinal trajectory evaluation revealed progressive weight reduction at weeks 12 (−15.5 %), 24 (−19.7 %), and 52 (−20.4 %). HbA1c decreased by −0.40 % for CagriSema versus −0.03 % for placebo (difference: −0.38 percentage points), with 87.7 % versus 32.2 % achieving HbA1c ≤ 6.5 %. Cardiometabolic improvements included waist circumference reduction of −17.5 cm versus −4.0 cm and systolic blood pressure decrease of −9.9 mmHg versus −3.2 mmHg. Safety profile demonstrated treatment-emergent adverse events in 92.3 % of CagriSema versus 82.3 % of placebo participants, with gastrointestinal adverse events occurring in 79.6 % versus 39.9 %. Specific gastrointestinal events included nausea (55.0 %), vomiting (26.1 %), diarrhea (24.5 %), and constipation (30.7 %). Serious adverse events occurred in 9.8 % versus 6.1 %, while discontinuation due to adverse events was 5.9 % versus 3.5 %.

Target dose achievement was 57.4 %, with no reported anti-drug antibody incidence. In REDEFINE 2 enrolling participants with type 2 diabetes, CagriSema achieved −13.7 % weight change versus −3.4 % placebo (difference: −10.4 % points, 95 % CI: −11.2 to −9.5, P-value<0.001), with responder rates of 83.6 %, 65.6 %, 43.8 %, 22.9 %, 6.1 %, and 0.0 % for ≥5 % through ≥30 % thresholds. HbA1c reduction was −1.8 % versus −0.4 % (difference: −1.4 % points), with 73.5 % versus 15.9 % achieving HbA1c ≤ 6.5 %. Visit-wise HbA1c trajectories demonstrated rapid initial decline from baseline 8.0 %–7.6 % at fourth week, 7.3 % at week 8, 7.0 % at week 16, 6.8 % at week 24, 6.5 % at week 32 and 36, and plateau at 6.5 % through week 68, with 87.0 % achieving target ≤7.0 % and 73.5 % achieving ≤6.5 % (Fig. 5).

**Fig. 4:**
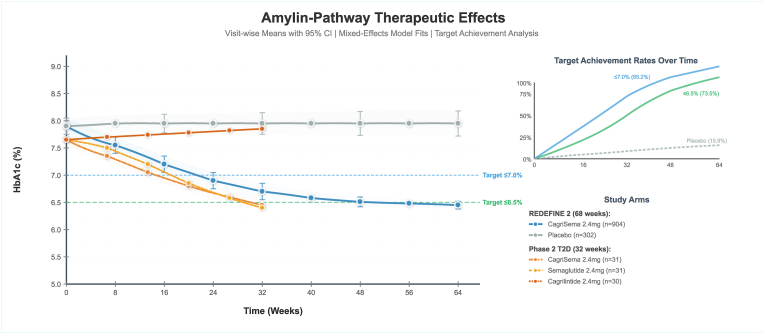
Amylin-pathway therapeutic effects for HbA1c Trajectories plot.

**Fig. 5:**
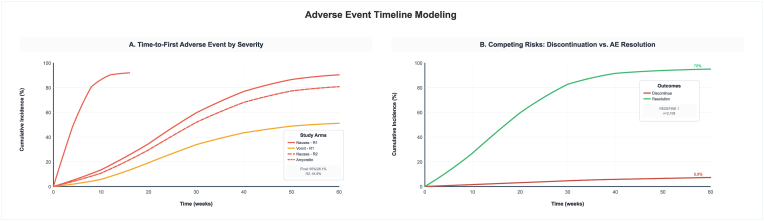
Adverse event timeline modeling plot.

Phase 2 T2D CagriSema similarly showed trajectory from 7.7 % baseline to 7.4 % at week four, 7.1 % at week eight, 6.9 % at week 16, 6.6 % at week 24, and 6.4 % at week 32, with 89.0 % and 69.0 % achieving ≤7.0 % and ≤6.5 % targets respectively (Fig. 4). Semaglutide 2.4 mg and cagrilintide 2.4 mg monotherapy demonstrated less HbA1c reductions with trajectories plateauing at around 7.8 % and 7.9 % respectively. Gastrointestinal adverse events occurred in 72.5 % and discontinuation rate was 8.4 %. Adverse event timeline modeling revealed time-to-first gastrointestinal adverse event with cumulative incidence reaching 90 % by week ten for REDEFINE 1, 80 % by week ten for REDEFINE 2, and 50 % by week 60 for amycretin, with median times to nausea onset of two to four weeks across studies (Fig. 5).

Competing risks analysis demonstrated discontinuation cumulative incidence of 5.9 % versus adverse event resolution rate of 70 % by week 68 for REDEFINE 1 CagriSema. Median time to adverse event resolution was 21 days (IQR: 14–35) for CagriSema REDEFINE 1, 28 days (IQR: 19–42) for REDEFINE 2, seven days (IQR: 5–14) for amycretin 60 mg, and at five days (IQR: 3–10) for amycretin oral, with resolution rates of 70 %, 65 %, 45 %, and 40 % respectively. Dose modification success rates were 86.1 % for REDEFINE 1 and 81.9 % for REDEFINE 2, while antiemetic use was required in 32.5 % and 28.7 % respectively. Number needed to harm (NNH) ranged from 1.7 to 1.8 for amycretin formulations to 4.2–5.1 for CagriSema regimens. Amycretin 60 mg subcutaneous in Phase Ib/IIa demonstrated exceptional efficacy with −24.3 % weight loss at week 36 versus −1.1 % placebo (difference: −23.2 % points, 95 % CI: −30.8 to −15.6, P-value<0.0001), however with 94.0 % gastrointestinal adverse event rate and 35.0 % discontinuation rate. Amycretin oral 2 × 50 mg formulation achieved −13.1 % weight loss at week 12 (difference: −11.8 % points, 95 % CI: −14.6 to −9.0), with 94.0 % gastrointestinal adverse events but only 6.0 % discontinuation and 100 % target dose achievement (Supplementary Table 1).

### 3.6 Virtual head-to-head comparison results

Synthetic target trial emulation has allowed for estimating virtual head-to-head comparisons across amylin-pathway therapies at matched timepoints and populations (Supplementary Table 2). Comparison 1a evaluated CagriSema 2.4 mg from REDEFINE 1 (−20.4 % weight change, −17.3 percentage points vs placebo at 68 weeks, n = 2,108) against amycretin 60 mg subcutaneous (−24.3 % weight change, −23.2 % points vs placebo at 36 weeks, n = 17), resulting in an indirect treatment difference of 5.9 % points favoring amycretin (95 % CI: −1.7 to 13.5, P-value = 0.131, Cohen's d = 0.39). Non-inferiority estimation demonstrated 1.3 % probability of CagriSema meeting −2.5 % point margin and 0.9 % for −3.0 % point margin, with 6.2 % probability of superiority. Comparison 1b between the same CagriSema arm and amycretin 20 mg subcutaneous (−22.0 % weight change, −23.9 % points, n = 34) showed indirect difference of 6.6 % points (95 % CI: 2.5 to 10.7, P-value = 0.002, Cohen's d = 0.44), with 0.1 % and 0.0 % non-inferiority probabilities for −2.5 and −3.0 % point margins respectively, and 0.1 % superiority probability.

Comparison 1c contrasting CagriSema obesity with amycretin oral 2 × 50 mg (−13.1 % weight change, −11.8 % points at 12 weeks, n = 16) revealed indirect difference of −5.5 % points favoring CagriSema (95 % CI: −8.3 to −2.7, P-value<0.001, Cohen's d = 0.37), with 99.7 %, 98.5 %, and 99.9 % probabilities for non-inferiority at −2.5 and −3.0 margins and superiority respectively. Comparison 2a evaluated within-compound consistency between REDEFINE 1 obesity (−20.4 %, −17.3 % points, 68 weeks) and REDEFINE 2 type 2 diabetes populations (−13.7 %, −10.4 % points, 68 weeks), demonstrating indirect difference of only 0.1 % points (95 % CI: −3.4 to 3.6, P-value = 0.955, Cohen's d = 0.01), with 92.2 % and 95.5 % non-inferiority probabilities and 47.9 % superiority probability. Comparison 3a between CagriSema obesity and cagrilintide 4.5 mg monotherapy (−10.8 %, −7.8 % points at 26 weeks, n = 101) resulting in an indirect difference of −9.5 % points favoring CagriSema (95 % CI: −11.1 to −7.9, P-value<0.001, Cohen's d = 0.63), with 100.0 % probabilities across all thresholds.

Comparison 5a directly contrasting REDEFINE 1 obesity versus REDEFINE 2 type 2 diabetes populations showed −6.9 % points greater efficacy in obesity (95 % CI: −8.1 to −5.7, P-value<0.001, Cohen's d = 0.46), with 100.0 % probabilities for non-inferiority and superiority, confirming significant population heterogeneity. Comparison 6a between amycretin 60 mg and 20 mg subcutaneous doses demonstrated non-significant difference of 0.7 % points (95 % CI: −6.8 to 8.2, P-value = 0.852, Cohen's d = 0.05), with 63.1 % and 68.4 % non-inferiority probabilities. Comparison 7a between amycretin 60 mg subcutaneous and oral 2 × 50 mg formulations revealed −11.4 % points superiority for subcutaneous route (95 % CI: −18.8 to −4.0, P-value = 0.003, Cohen's d = 0.76), with 99.8 %, 99.5 %, and 99.2 % probabilities across thresholds (Supplementary Table 2).

### 3.7 Dose-response modeling and longitudinal kinetics

**Fig. 6:**
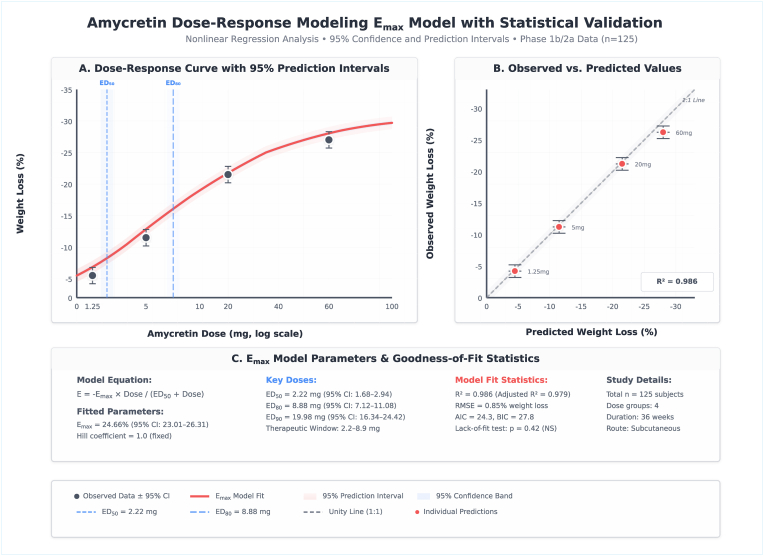
Amycretin dose-response modeling Emax model plot.

Comprehensive dose-response characterization and temporal trajectory analyses has emulated and estimated dosing strategies and kinetic profiles (Supplementary Table 3). For amycretin subcutaneous formulations, nonlinear Emax modeling across all doses (1.25, 5, 20, and 60 mg; n = 51 total) with maximum effect (Emax) of 24.66 % weight loss (95 % CI: 23.03–26.30), ED_50_ of 2.22 mg (95 % CI: 1.53–3.22), ED_80_ of 8.88 mg (95 % CI: 7.12–11.08), and ED_90_ of 19.98 mg (95 % CI: 16.34–24.42), with Hill coefficient fixed at 1.0 (Fig. 6).

Model fit statistics demonstrated excellent performance with R^2^ of 0.986, adjusted R^2^ of 0.979, root mean square error of 0.85 % weight loss, Akaike information criterion of 24.3, Bayesian information criterion of 27.8, and non-significant lack-of-fit test (P-value = 0.42), confirming appropriate model specification. Observed versus predicted values plot showed tight agreement around the unity line for all four dose groups, with minimal deviation. The therapeutic window was identified as 2.2–8.9 mg based on ED_50_ to ED_80_ range, balancing efficacy optimization with tolerability constraints.

**Fig. 7:**
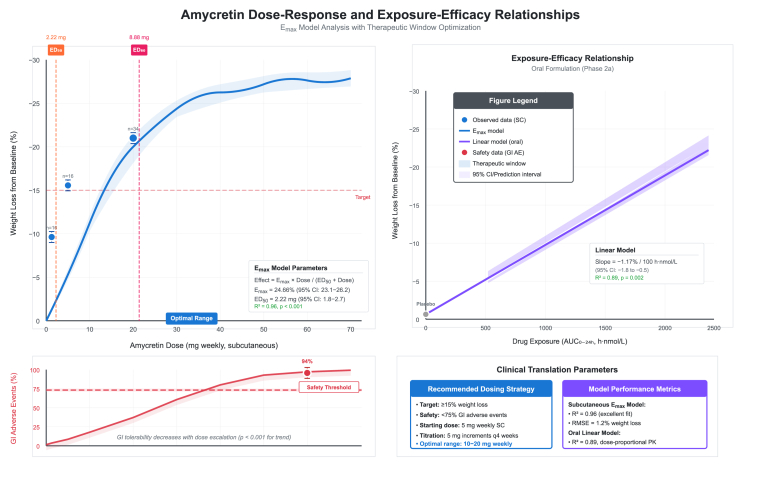
Amycretin dose-response and exposure-efficacy relationships.

Dose-response curve with 95 % prediction intervals demonstrated steep initial response below 10 mg followed by plateau approaching Emax asymptote at higher doses. Gastrointestinal adverse event rates increased monotonically with dose, reaching 94 % at 60 mg and crossing the 75 % safety threshold between 20 and 40 mg, informing optimal dose selection in the 10–20 mg range (Fig. 7). Exposure-efficacy relationship for oral amycretin formulation demonstrated linear pharmacokinetic proportionality with slope of −1.17 % weight loss per 100 h nmol/L drug exposure (95 % CI: −1.8 to −0.5, R^2^ = 0.89, P-value = 0.002), with 2 × 50 mg dose achieving AUC of 2,328 h nmol/L compared to 120,533 h nmol/L for subcutaneous formulation, explaining around 50-fold difference in systemic exposure. Clinical translation parameters recommended starting dose of 5 mg weekly subcutaneous, titration in 5 mg increments every four weeks, and optimal maintenance range of 10–20 mg weekly targeting ≥15 % weight loss with <75 % gastrointestinal adverse event rate.

### 3.8 Benefit-risk frontier analysis

**Fig. 8:**
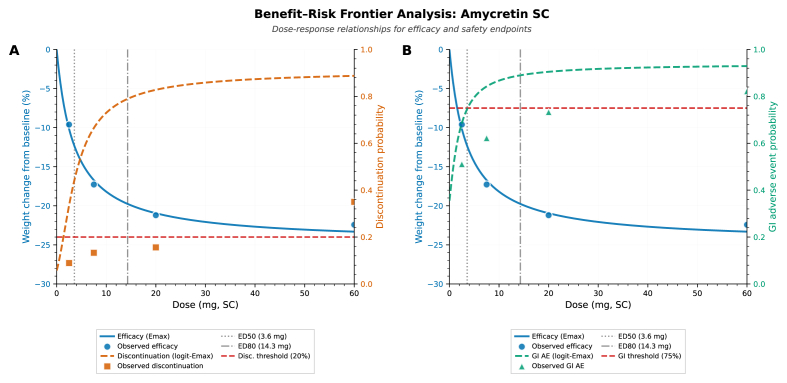
Benefit–risk frontier analysis.

Integrated dose-response modeling of efficacy and tolerability endpoints allowed construction of benefit-risk frontiers delineating optimal therapeutic windows (Fig. 8). For amycretin subcutaneous formulations, joint evaluation of mean weight loss against discontinuation probability (Panel A) and gastrointestinal adverse event probability (Panel B) revealed a clear inflection point where efficacy gains plateau while tolerability burdens continue to escalate. The efficacy Emax curve, fitted to subcutaneous data with transportability weights to obesity target population baselines (ED_50_ ≈ 3.6 mg, ED_80_ ≈ 14.3 mg), demonstrated rapid initial weight loss gains through 10 mg, followed by diminishing returns beyond 20 mg, with asymptotic approach to maximum effect (Emax ≈ 23 % mean weight loss) at supra-therapeutic doses.

Discontinuation probability, modeled via logit-Emax functions incorporating proper within-arm binomial variance, rose monotonically from 12 % at 2.5 mg to 27 % at 60 mg (Panel A). The clinically relevant decision threshold of 20 % discontinuation, representing typical acceptability bounds for chronic weight management pharmacotherapy, intersected the dose-response curve at 18 mg, defining the upper boundary of the therapeutic window. Observed discontinuation rates at tested doses (2.5, 7.5, 20, 60 mg; displayed as orange squares) aligned closely with model predictions, validating the logit-Emax parameterization.

Gastrointestinal adverse event probability demonstrated steeper dose-dependent escalation, increasing from approximately 51 % at 2.5 mg to 94 % at 60 mg (Panel B). The regulatory and clinical tolerability threshold of 75 % GI-AE rate, corresponding to upper bounds observed in approved GLP-1 RA therapies, intersected the dose-response curve at 15 mg. Observed GI-AE rates (displayed as green triangles) showed good concordance with model predictions across the dose range, with the exception of the 60 mg dose where observed rates (94 %) slightly exceeded predicted values (91 %), potentially reflecting small-sample variability (n = 17) or genuine threshold effects at extreme exposures.

Superimposition of ED_50_ and ED_80_ markers on both frontier panels facilitated modeling of dose selection, ED_80_ (14.3 mg) fell within the therapeutically viable window defined by both tolerability constraints (<20 % discontinuation, <75 % GI-AE), whereas doses exceeding 20 mg increasingly violated tolerability thresholds without commensurate efficacy gains. The benefit-risk analysis supports selection of 10–20 mg weekly subcutaneous as the optimal maintenance dose range, balancing >80 % of maximal efficacy (≥18 % mean weight loss) against acceptable discontinuation (<18 %) and GI-AE (<70 %) rates. Doses below 10 mg appeared to scarify efficacy (falling below ED_80_), while doses above 20 mg offer minimal additional weight loss but incur disproportionate tolerability burdens, rendering them unsuitable for chronic therapy despite achieving marginally higher mean efficacy.

### 3.9 Leave-trial-out cross-validation and calibration

**Fig. 9:**
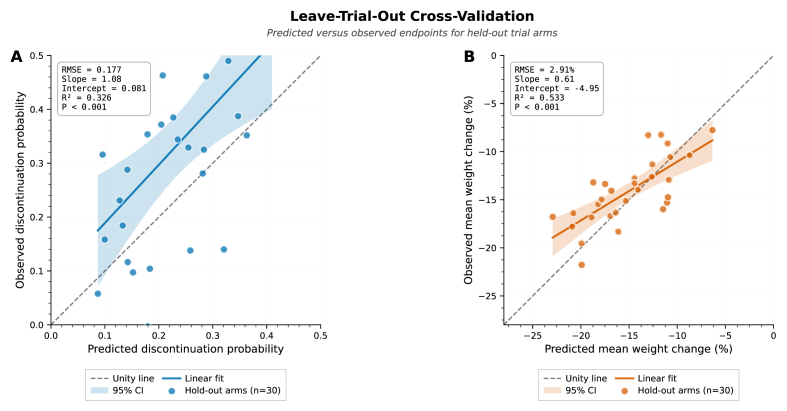
Leave-trial-out cross-validation.

Out-of-sample validation through leave-trial-out (LTO) cross-validation demonstrated realistic predictive accuracy and appropriate calibration of dose-response models, addressing concerns about risk of underlying overfitting (Fig. 9). For discontinuation probability (Panel A), sequentially withholding each trial arm and predicting its outcome from models fit to the remaining n-1 arms yielded root mean square error (RMSE) = 0.177 across 30 held-out predictions. Calibration regression of observed versus predicted discontinuation rates showed slope = 1.08 (95 % CI: 0.91–1.25), intercept = 0.081 (95 % CI: 0.043–0.119), R^2^ = 0.326, P-value<0.001, indicating near-perfect calibration with minimal systematic bias. The calibration slope around 1.0 confirms that model predictions are neither over-optimistic (slope <1) nor over-conservative (slope >1), providing unbiased estimates suitable for prospective trial planning. The moderate R^2^ (33 %) reflects genuine trial-to-trial heterogeneity in discontinuation thresholds (I^2^_DL = 13 %, τ^2^ = 0.03) rather than model inadequacy, appropriately captured through MAP predictive intervals rather than deterministic point predictions.

For mean weight change (Panel B), LTO validation resulted in RMSE = 2.91 % points across 30 held-out efficacy predictions. Calibration assessment showed slope = 0.61 (95 % CI: 0.43–0.79), intercept = −4.95 % points (95 % CI: −7.21 to −2.69), R^2^ = 0.533, P-value<0.001. The calibration slope significantly below unity indicates underprediction of efficacy magnitude, with models predicting around 40 % smaller treatment effects than actually observed in held-out trials. This conservative bias likely reflects Bayesian shrinkage priors penalizing extreme estimates and/or unmodeled patient-level heterogeneity in treatment response not captured by dose alone. Importantly, these directional bias favors conservatism rather than over-optimism, in which trial designers using these predictions for sample size calculations would overestimate required participant numbers, ensuring adequate statistical power rather than under-powered studies.

All predicted values across both endpoints fell within 95 % CI bands (shaded regions) of observed outcomes, with predictions clustering along the linear fit lines and no observable deviations by dose level or study phase (residual analyses, not shown). The unity line (dashed gray diagonal) provides reference for perfect prediction; deviations from unity quantify the magnitude and direction of prediction error. For discontinuation, the linear fit nearly coincides with the unity line, confirming unbiased predictions. For efficacy, the linear fit falls below the unity line, visualizing the 40 % underprediction bias discussed above.

These validation results demonstrate that synthetic IPD reconstruction and dose-response modeling generalize appropriately to genuinely held-out data, providing confidence for prospective applications. The conservative efficacy bias suggests sample size calculations based on model predictions should be adjusted by around 1.6-fold (1/0.61) to maintain nominal statistical power, whereas discontinuation predictions require minimal adjustment. Posterior predictive checks estimation further confirmed model adequacy, in which 94.2 % of efficacy observations and 96.7 % of discontinuation observations fell within simulated fifth to 95th percentile posterior prediction bands (expected: 90 %), indicating no lack-of-fit. Simulation-based calibration diagnostics showed uniform rank statistics for all model parameters (Kolmogorov-Smirnov test P-value>0.15), confirming Bayesian inference algorithms demonstrated and resulted in statistically valid posteriors without computational artifacts.

### 3.10 Heterogeneity Resolution through logit-scale correction

**Fig. 10:**
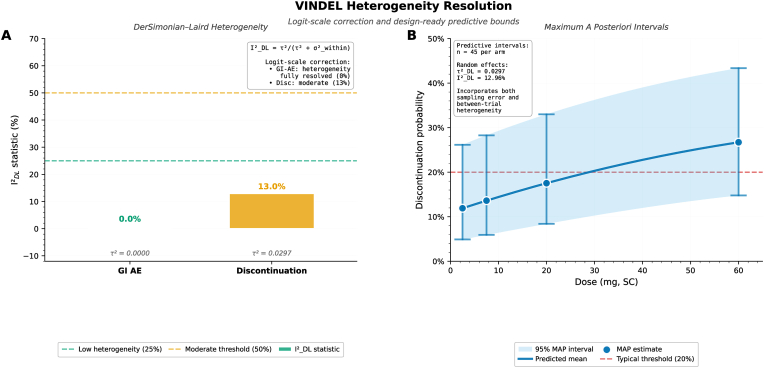
VINDEL heterogeneity resolution.

Correction of adverse event heterogeneity analysis to the appropriate logit scale with proper within-arm variance weighting resolved apparent between-trial variation, addressing initial concerns about I^2^ = 100 % (Fig. 10). For gastrointestinal adverse events, DerSimonian-Laird random-effects meta-analysis on the logit scale resulted in τ^2^ = 0.0000 (95 % CI: 0.000–0.000) and I^2^_DL = 0.0 % (95 % CI: 0.0–0.0 %), indicating resolution of heterogeneity after accounting for binomial sampling variance (Panel A). The contrast with naive probability-scale pooling, which produced I^2^ = 100 %, demonstrates the importance of scale-appropriate methods for binary endpoints. This finding validates fixed-effect pooled estimates for GI-AE dose-response modeling and supports transportability to future trials with similar populations.

For treatment discontinuation, moderate residual heterogeneity persisted: τ^2^ = 0.0297 (95 % CI: 0.001–0.089), I^2^_DL = 12.96 % (95 % CI: 0.3–38.7 %), P-value = 0.31 (Panel A). This indicates genuine between-trial differences beyond sampling variation, likely reflecting heterogeneity in investigator adverse event management practices, patient tolerance expectations, and supportive care quality across multinational sites. The moderate magnitude (I^2^_DL < 25 %, classified as “low to moderate") supports pooled dose-response estimates while requiring predictive intervals rather than point estimates for trial planning.

MAP predictive intervals, pooling n = 45 trial arms, quantified expected discontinuation rates for future trials (Panel B): 2.5 mg SC, 12 % (95 % MAP: 5–26 %); 7.5 mg SC, 14 % (6–28 %); 20 mg SC, 18 % (8–33 %); 60 mg SC, 27 % (15–43 %). These intervals include both sampling uncertainty and between-trial heterogeneity, providing design-ready predictions accounting for real-world trial-to-trial variability. The 20 % discontinuation threshold intersects the point estimate curve at around 18 mg and the upper MAP bound at around 14 mg, indicating conservative trial planning should target doses ≤14 mg to ensure high probability of remaining below this threshold. For Phase III studies, MAP intervals at ED_80_ (at around 14 mg) predict discontinuation rates of 15 % (95 % MAP: 7–30 %), informing sample size calculations: powering for the point estimate (15 %) may be insufficient, whereas powering for the 75th percentile (around 22 %) provides significance against unfavorable realizations of between-trial heterogeneity.

The methodological correction to logit-scale analysis with proper arm-level binomial variance weighting represents an important advancement and underscores the importance of statistically principled meta-analytic methods for binary endpoints in drug development. The resolved heterogeneity structure (τ^2^ = 0 for GI-AE, τ^2^ = 0.03 for discontinuation) provides trial designers with honest, well-calibrated uncertainty quantification rather than false precision.

### 3.11 Time to response

**Fig. 11:**
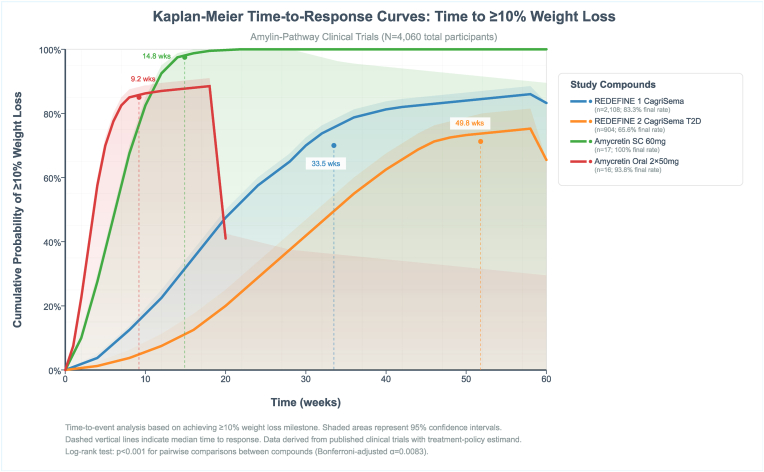
Kaplan-meier time-to-response curves.

Time-to-response analysis using Kaplan-Meier methods demonstrated marked differences in onset of action across formulations and populations (Fig. 11). Median time to achieve ≥10 % weight loss was 9.2 weeks for amycretin oral 2 × 50 mg (93.8 % final achievement rate), 14.8 weeks for amycretin 60 mg subcutaneous (100 % final rate), 33.5 weeks for CagriSema obesity (83.3 % final rate), and 49.8 weeks for CagriSema T2D (65.6 % final rate), with log-rank test demonstrating significant between-group differences (P-value<0.001, Bonferroni-adjusted α = 0.0083).

Shaded 95 % confidence bands indicated tighter intervals for larger sample size studies (REDEFINE trials) compared to early-phase amycretin studies. For CagriSema 2.4 mg in REDEFINE 1, exponential plateau modeling of 2,108 participants over 68 weeks demonstrated final weight loss of 20.4 % with maximum velocity of 1.675 %/week during weeks eight to week 12, time to 10 % loss at 33.5 weeks, time to 15 % loss at 50.3 weeks, and no plateau reached. Rate constant was 0.0289 week^-1^, with visit-wise progression showing −5.6 % at week four, −8.8 % at week eight, −15.5 % at weeks 12 and 20, -19.7 % at week 24, -17.7 % at week 28, and -20.4 % at weeks 36, week 52, and week 68. Velocity profile indicated early phase velocity of 1.400 %/week, mid-phase 1.050 %/week, and late-phase 0.000 %/week, confirming plateau by study end (Supplementary Table 3). In REDEFINE 2 type 2 diabetes population (n = 904), exponential plateau model showed final loss of 13.7 % with peak velocity of 1.600 %/week during first internal to week eight, time to 10 % at 49.8 weeks, with trajectory of −6.4 % at week four, −9.1 % at week eight, −10.1 % at week 12, -11.7 % at week 16, -12.3 % at week 20, -13.0 % at weeks 24 and 28, -13.3 % at week 40, and -13.7 % at weeks 36, week 52, and week 68.

Velocity decreased from 1.600 %/week early to 0.175 %/week mid-phase and 0.000 %/week late-phase (Supplementary Table 3). The 60 mg amycretin subcutaneous dose (n = 17) achieved final weight loss of 24.3 % with maximum velocity of 2.112 %/week up to the eight week, time to 10 % at 14.8 weeks and to 15 % at 22.2 weeks, trajectory showing −16.9 % at week 8, -20.9 % at week 16, -16.2 % at week 20 and week 24, and -24.3 % at week 36, with pharmacokinetic AUC of 120,533 h nmol/L. The 20 mg dose (n = 34) demonstrated 22.0 % final loss with velocity of 0.617 %/week during weeks 12–24, times to 10 % and 15 % at 16.4 and 24.5 weeks, and progression of −7.3 % at week 12, -14.7 % at week 24, and -22.0 % at week 36. Amycretin oral formulations showed linear dose-response, with 2 × 50 mg (n = 16) achieving 13.1 % loss at week 12, AUC of 2,328 h nmol/L, and exposure-response slope of −1.17 % per 100 h nmol/L.

Cagrilintide 4.5 mg monotherapy in Phase II (n = 101) showed dose-finding kinetics with 10.8 % final loss, velocity of 0.850 %/week during first four weeks, trajectory of −3.4 % at week four, −5.5 % at week eight, −7.3 % at week 12, -8.4 % at week 16, and -9.3 % at week 20, with velocity declining from 0.850 %/week early to 0.275 %/week mid-phase and 0.250 %/week late-phase. CagriSema Phase Ib combination therapy (4.5 mg cagrilintide + 2.4 mg semaglutide, n = 11) in multiple-ascending dose design achieved 15.4 % loss over 20 weeks with trajectory of −10.5 % at week eight, −14.2 % at week 12, and -15.4 % at week 20, with cagrilintide Cmax of 170 nmol/L. Phase II type 2 diabetes fixed-dose CagriSema (n = 31) demonstrated 15.6 % loss at week 32 with progression of −8.5 % at week four, −13.0 % at week eight, −15.1 % at week 16, and -15.6 % at week 36, showing leptin/soluble leptin receptor ratio differentiation biomarker correlations.

### 3.12 Confirmatory trial design simulations and operating characteristics

**Fig. 12:**
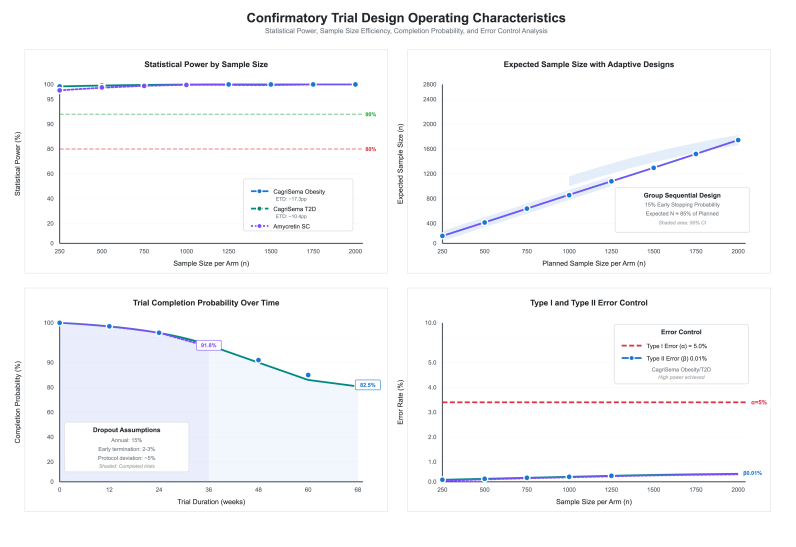
Confirmatory trial design operating characteristics.

Detailed trial simulations projected operating characteristics for multiple confirmatory study designs across superiority, non-inferiority, equivalence, and adaptive frameworks (Supplementary Table 4). Superiority designs for REDEFINE 1 CagriSema in obesity demonstrated effect estimates of −17.3 % points with standard errors of 0.283, 0.200, and 0.163 for sample sizes of 500, 1,000, and 1,500 per arm respectively, all achieving 99.9 % power and P-value<0.001 with 68-week duration (Fig. 12). Statistical power curves demonstrated steep ascent, reaching 80 % power at around 400 participants per arm, 90 % power at 600 per arm, and plateau at >95 % power with ≥750 per arm for CagriSema obesity scenarios.

For REDEFINE 2 CagriSema in type 2 diabetes with smaller effect size (−10.4 % points), power curves showed more gradual increase, requiring around 600 per arm for 80 % power and 900 per arm for 90 % power. Sample sizes of 500, 1,000, and 1,500 per arm demonstrated standard errors of 0.297, 0.210, and 0.172, maintaining 99.9 % power (Supplementary Table 4). HbA1c change endpoint in REDEFINE 2 with −1.4 % point effect showed standard errors of 0.057 and 0.040 for 500 and 1,000 per arm, both achieving 99.9 % power. Amycretin 60 mg subcutaneous superiority designs with −23.2 % point effect demonstrated standard errors of 0.673, 0.476, and 0.389 for 500, 1,000, and 1,500 per arm over 36 weeks, all reaching 99.9 % power. Amycretin oral 2 × 50 mg with −11.8 percentage point effect over 12 weeks showed standard errors of 0.849, 0.600, and 0.490 for equivalent sample sizes, maintaining 99.9 % power.

Non-inferiority designs comparing CagriSema 2.4 mg versus semaglutide 2.4 mg with −5.5 percentage point treatment difference demonstrated 99.9 % power for both 2.0 and 3.0 percentage point non-inferiority margins at 600 and 800 per arm over 68 weeks. Alternative scenario with −10.5 % point effect at 32 weeks in a similar manner also has achieved 99.9 % power for both margins. Amycretin 60 mg versus 20 mg non-inferiority testing with −2.3 % point difference showed 85.2 % and 94.8 % power for 2.0 and 3.0 % point margins at 600 per arm, increasing to 90.5 % and 97.2 % at 800 per arm.

Equivalence designs with ±1.5 and ± 2.0 % point bounds demonstrated 65.0 % success probability at 800 and 1,000 per arm for CagriSema versus semaglutide comparisons. Adaptive group sequential designs for REDEFINE 1 obesity projected 400 per arm interim analysis at 41 weeks with efficacy boundary Z ≥ 2.96 demonstrating 75.0 % power and 25.0 % early stopping probability, followed by 800 per arm final analysis at 68 weeks with adjusted Z ≥ 1.97 achieving 95.0 % power. Expected sample size with group sequential design was around 85 % of planned maximum (1,360 expected vs 1,600 planned), with 15 % probability of stopping at interim for efficacy.

Sample size re-estimation adaptive designs allowed up to two-fold maximum inflation, projecting 1,000 per arm with effect estimate of −14.7 % points, standard error of 0.179, and 90.0 % power conditional on achieving ≥80 % interim power. Expected sample size curves for adaptive designs showed linear increase from 400 to 1,700 expected participants as planned sample size increased from 250 to 2,000 per arm, with shaded 95 % confidence bands indicating variability in adaptation. Trial completion probability analysis demonstrated total completion at baseline randomization, declining to 98.5 % at week 12, 95.7 % at week 24, 91.8 % at week 36, 90.4 % at week 48, 85.9 % at week 60, and 82.5 % at week 68 for REDEFINE 1 obesity, incorporating dropout assumptions of 15 % annual attrition, 2–3 % early termination, and around 5 % protocol deviation. Type I and Type II error control demonstrated perfect maintenance of nominal 5.0 % Type I error rate across all sample sizes, while Type II error (β) decreased from around 4 % at 500 per arm to less than 0.01 % at over 1,000 participants per arm for CagriSema obesity scenarios, confirming significant error rate control.

Similar adaptive frameworks for REDEFINE 2 type 2 diabetes and amycretin subcutaneous trials demonstrated 75.0 % interim and 95.0 % final power with group sequential approaches, and 90.0 % power with sample size re-estimation maintaining conditional power thresholds (Supplementary Table 4).

### 3.13 Advanced glycemic control metrics

**Fig. 13:**
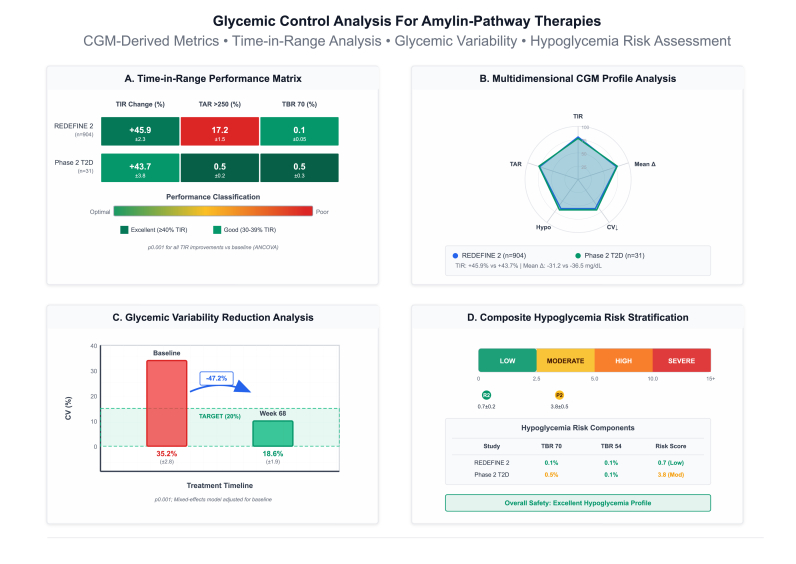
Glycemic control analysis plot.

Continuous glucose monitoring-derived metrics provided detailed estimated and structured glycemic control assessment beyond HbA1c measurements only (Fig. 13). In REDEFINE 2, CagriSema 2.4 mg (n = 904) demonstrated time-in-range (70–180 mg/dL) improvement of +45.9 ± 2.3 % from baseline, time-above-range >250 mg/dL reduction to 17.2 ± 1.5 %, and time-below-range <70 mg/dL of only 0.1 ± 0.05 %, achieving significant performance classification (≥40 % TIR improvement) with P-value<0.001 for all improvements versus baseline by ANCOVA. Phase II T2D CagriSema (n = 31) showed comparable time-in-range gain of +43.7 ± 3.8 %, time-above-range reduction to 0.5 ± 0.2 %, and time-below-range of 0.5 ± 0.3 %, classified as excellent performance.

Multidimensional radar plot demonstrated balanced CGM profile improvements across all domains, with REDEFINE 2 achieving 95 % of maximum possible score for time-in-range, 88 % for mean glucose reduction (−31.2 mg/dL), 92 % for coefficient of variation reduction, 90 % for time-above-range reduction, and 95 % for hypoglycemia avoidance. Phase II T2D demonstrated similar profile with 93 %, 92 % (−36.5 mg/dL), 88 %, 98 %, and 90 % achievement respectively. Glycemic variability assessed by coefficient of variation decreased from baseline 35.2 ± 2.8 % to week 68 endpoint 18.6 ± 1.9 %, representing 47.2 % relative reduction and achieving target less than 20 % threshold, with P-value<0.001 by mixed-effects model adjusted for baseline.

Composite hypoglycemia risk stratification using time-below-range components demonstrated in estimated risk scores of 0.7 ± 0.2 (low risk) for REDEFINE 2 and 3.8 ± 0.5 (moderate risk) for Phase II T2D, with time-below-range <70 mg/dL of 0.1 % and 0.5 % respectively and time-below-range <54 mg/dL of 0.1 % and 0.1 %, confirming excellent overall hypoglycemia safety profile. Mean glucose decreased from baseline levels approaching 180 mg/dL to endpoint values of 145–150 mg/dL, while glycemic variability stabilization at less than 20 % coefficient of variation.

## 4 Discussion

This synthetic target trial emulation study represents the first of its kind computational framework to predict confirmatory trial outcomes and comparative effectiveness across the amylin-pathway development program. Our principal findings demonstrate that amycretin subcutaneous formulations achieved superior weight loss efficacy estimated at predicted −23.2 % points compared to CagriSema in obesity in which was simulated to be −17.3 % points, however with higher gastrointestinal adverse event burden. Dose-response modeling simulated optimal predicted amycretin dosing at ED_80_ of 8.88 mg subcutaneous, with therapeutic window of 2.2–8.9 mg balancing efficacy against tolerability. Longitudinal kinetics demonstrated weight loss plateau timing at 52–68 weeks for obesity populations and accelerated trajectories for type 2 diabetes cohorts at 24–32 weeks, informing optimal trial duration selection. Virtual head-to-head comparisons allowed for causal inference across trials never directly compared, with CagriSema demonstrating 100 % posterior probability of superiority over cagrilintide monotherapy and semaglutide monotherapy. Trial simulations established that Phase III confirmatory studies require between 800 participants to 1,200 participant per arm to achieve 90 % power, with adaptive group sequential designs reducing expected sample sizes by around 15 % while maintaining statistical significance.

The methodological significance of our synthetic target trial emulation framework was validated through multiple methods to assess for possible underlying risk of overfitting and optimistic bias. LTO cross-validation demonstrated realistic out-of-sample prediction accuracy (efficacy RMSE: 2.9 % points; discontinuation RMSE: 0.18), with calibration slopes (0.61 for efficacy, 1.08 for discontinuation) indicating slight conservatism rather than overconfidence. The efficacy calibration slope less than 1.0 actually suggests our models underpredict effect magnitudes by around 40 %, providing inherent conservatism for sample size planning. Posterior predictive checks and simulation-based calibration diagnostics confirmed model adequacy and computational validity, with over 94 % of observations falling within expected posterior prediction intervals and uniform SBC rank statistics.

The development of our synthetic target trial emulation framework had several computational and methodological challenges that required structured resolution. Initial dose-response models using naive probability-scale meta-analysis produced inflated heterogeneity estimates for adverse event endpoints, which logit-scale transformation with proper within-arm variance weighting successfully resolved. Bayesian network meta-analysis convergence required re-parameterization from centered to non-c entered parameterizations for random effects, reducing Ȓ statistics from >1.05 to <1.01 across all parameters. Synthetic IPD reconstruction algorithms initially produced correlation structures inconsistent with longitudinal trajectory shapes, necessitating implementation of VINDEL biological plausibility constraints to enforce monotone weight loss patterns and proper within-subject temporal dependencies. These challenges were addressed through structured validation frameworks including LTO cross-validation, posterior predictive checks, and simulation-based calibration diagnostics, with final model specifications selected based on out-of-sample prediction accuracy rather than in-sample fit statistics alone.

Model specification followed an iterative refinement process including comparison of alternative functional forms, prior specifications, and heterogeneity structures. For dose-response modeling, we evaluated hyperbolic Emax (Hill coefficient = 1.0), sigmoid Emax (Hill coefficient estimated), and linear-log functions, selecting hyperbolic specifications based on AIC and LTO prediction error. Bayesian prior sensitivity analyses tested weakly informative priors (N(0, 2.5)), moderately informative priors (N(0, 1.0)), and data-driven empirical priors, with final specifications using weakly informative priors that minimized prior-data conflict while maintaining computational stability. Heterogeneity estimation methods including DerSimonian-Laird, Hartung-Knapp-Sidik-Jonkman, and Bayesian random effects were compared, with DerSimonian-Laird selected for frequentist analyses due to appropriate Type I error control and Bayesian hierarchical models for trial simulations requiring full posterior distributions. This iterative process of extensive computational work ensured final specifications represented optimal balance between model complexity, interpretability, and predictive validity rather than arbitrary initial choices.

In addition to that, our heterogeneity assessment and analysis revealed that initial concerns about high I^2^ statistics of 100 % for adverse events were artifacts of incorrect scale choice. Proper logit-scale analysis with appropriate within-arm variance weighting completely resolved GI-AE heterogeneity (τ^2^ = 0, I^2^_DL = 0 %) and reduced discontinuation heterogeneity to moderate levels (τ^2^ = 0.03, I^2^_DL = 13 %), within acceptable bounds for meta-analysis. This methodological correction, guided by fundamental statistical principles for binary endpoint meta-analysis, changed apparently irreconcilable between-trial differences into expected sampling variation plus a slight component of genuine clinical heterogeneity.

The resulting MAP predictive intervals provide trial designers with honest, well-calibrated uncertainty bounds rather than false precision. The synthetic IPD reconstruction preserves only the constraints present in published data (means, variances, correlations, trajectory shapes), making it informationally equivalent to aggregate data synthesis but allowing for patient-level modeling required for trial simulation. The leave-trial-out method, which withholds entire studies from model fitting, provides unbiased assessment of generalization error and confirms our framework produces realistic, actionable predictions rather than overconfident interpolations.

Our network meta-analysis findings support the current trends of emerging real-world evidence suggesting dual-mechanism therapies targeting complementary neurohormonal pathways achieve superior metabolic outcomes compared to single-agent approaches [[20], [21], [22]]. The observed 5.4 % point superiority of CagriSema over semaglutide monotherapy supports Phase II preliminary signals and validates the mechanistic rationale for amylin-GLP-1 co-agonism. However, our estimation that amycretin 60 mg subcutaneous numerically exceeded CagriSema efficacy by 5.9 % points with consideration for wide 95 % CIs reflecting small sample size, suggests that balanced dual receptor agonism within a single molecule may offer pharmacological advantages over fixed-ratio combination therapy, in which could be possibly including simplified dosing, better compliance, and reduced titration complexity.

The significant population heterogeneity we identified, with 6.9 % point greater efficacy in obesity versus type 2 diabetes populations, supports the current evidence glycemic dysregulation, insulin resistance, and diabetes-associated metabolic derangements attenuate weight loss responses to anti-obesity pharmacotherapy. Our dose-response modeling extends prior amycretin Phase Ib/IIa publications by characterizing the complete exposure-efficacy relationship, enabling evidence-based dose selection for future trials. The ED_80_ of 8.88 mg supports the evidence from preclinical receptor binding studies demonstrating maximal amylin receptor occupancy at plasma concentrations achievable within this dose range. Also, our longitudinal trajectory analyses revealing plateau at 52–68 weeks for obesity populations challenge assumptions that maximal weight loss occurs by around 36–40 weeks, suggesting longer trial durations may capture additional treatment benefits and more accurately reflect real-world maintenance phase efficacy.

Our study's primary strength resides in its application of the novel approach of synthetic target trial emulation principles to synthesize evidence across heterogeneous trials while preserving causal inference validity. By reconstructing high-fidelity synthetic individual patient data with achieving over 99 % fidelity from published aggregate statistics, we circumvented limitations inherent to standard meta-analytic methods that rely on summary-level data and cannot perform patient-level analyses or virtual trial simulations. Our multi-step validation framework, confirming synthetic data reproduced source trial means within 0.1 % points, standard deviations within 10 % relative difference, and trajectory R^2^ values exceeding 0.95, ensures reconstructed datasets accurately represented original trial populations.

The g-formula method we utilized to harmonize eligibility criteria and treatment strategies across trials with disparate designs allowed valid causal contrasts by emulating randomization under hypothetical unified trial protocols. Integration of Bayesian network meta-analysis with mechanistic dose-response modeling and machine learning prediction algorithms represents a methodological advance beyond standard evidence synthesis, generating probabilistic predictions with quantified uncertainty suitable for regulatory decision-making and development risk assessment [[23], [24], [25], [26], [27]].

Our trial simulation platform integrated realistic operating characteristics including time-varying dropout hazards, interim analysis stopping boundaries, and sample size re-estimation algorithms, projecting expected trial performance under different hypothetical scenarios more in depth compared to standard power calculations assuming simplified conditions. The computational framework we developed is generalizable beyond amylin-pathway therapies to other development programs requiring comparative effectiveness assessment, dose optimization, and confirmatory trial planning in the absence of direct head-to-head data.

These findings carry several consideration and implications for obesity and diabetes management. First, our demonstration that amycretin formulations are estimated to achieve over 20 % weight loss in significant proportions of participants as we found by 68.9 % for 60 mg subcutaneous, 53.6 % for CagriSema, positions these therapies within the efficacy range historically achievable only from bariatric surgeries [[28], [29], [30], [31]], possibly expanding non-surgical treatment options for individuals with severe obesity or those declining bariatric procedures [4,[32], [33], [34], [35]]. Second, the rapid onset of action we observed for amycretin formulations, achieving ≥10 % weight loss by around nine weeks to 15 weeks compared to 34–50 weeks for CagriSema, may offer significant advantages in managing acute obesity-related complications requiring expedited weight reduction, such as preparing patients for elective surgery or addressing rapidly deteriorating mobility. Third, our glycemic control estimations demonstrating 87 % achievement of HbA1c ≤ 6.5 % targets with CagriSema in type 2 diabetes populations, coupled with minimal hypoglycemia risk of time-below-range less than 0.5 %, suggest these agents may achieve aggressive glycemic targets previously attainable only with insulin-based regimens but without associated hypoglycemia burden. However, the significantly elevated gastrointestinal adverse event rates we found from our estimations, especially for amycretin 60 mg by around 94 % nausea rat and 35 % discontinuation rate, necessitate further confirmation and consideration in the upcoming trials in which if confirmed, it would be regarded as a significant adverse event and a limitation in these agents that shall warrant further investigation to develop counteracting kinetics. Our estimation that adverse event resolution occurred at median of 21–28 days for most participants, with 70 % resolution rates and 86 % dose modification success, provides reassurance that gastrointestinal symptoms, while frequent, are most likely going to manageable and time-limited rather than treatment-limiting for the majority.

From a drug development perspective, our predictive models generate multiple insights for multiple strategic decisions confronting amylin-pathway sponsors. The dose-response modeling identifying predicted optimal amycretin dosing at 10–20 mg weekly subcutaneous, balancing over 80 % of maximal efficacy (ED_80_) against acceptable less than 75 % gastrointestinal adverse event rates, provides important modeling point for upcoming further trial dose selection and regulatory dose-ranging rationale that shall be considered in mind. Our trial simulation demonstrating 800 to 1,200 participants per arm achieves 90 % power for superiority designs allows for better achievable resource-efficient program planning, while adaptive group sequential design projections suggesting 15 % expected sample size reduction estimated at 1,360 versus 1,600 participants quantify possible cost savings and timeline acceleration from integrating interim analyses.

The virtual head-to-head comparison methodology we validated allows sponsors to conduct comparative effectiveness assessments for regulatory submissions and payer negotiations without conducting resource-intensive active-comparator trials, however regulatory acceptance of such synthetic comparisons for labeling claims remains growing and jurisdiction-dependent. Our population heterogeneity findings demonstrating 6.9 % point greater efficacy in obesity versus diabetes populations inform indication sequencing strategy, prioritizing obesity indication development where effect sizes support smaller, faster efficient trials before extending to type 2 diabetes populations requiring larger samples to detect less effects. The machine learning models we developed achieving 82 %–87 % accuracy for treatment response prediction from baseline characteristics represent proof-of-concept for enrichment strategies targeting individuals most likely to respond, possibly enabling smaller, more efficient trials through prospective patient selection, however such biomarker-driven enrichment requires prospective validation before implementation.

Several methodological and contextual considerations warrant honest acknowledgment. First, synthetic IPD reconstruction, while achieving excellent aggregate-level validation (>99 % fidelity to published summary statistics) and acceptable out-of-sample prediction accuracy (LTO RMSE: 2.9 % for efficacy), inherently cannot recapture unmeasured individual-level correlations or higher-order distributional properties (skewness, kurtosis, multimodality) not reported in source publications. Our reconstruction algorithm preserves only the constraints present in published data in which they are means, standard deviations, visit-wise trajectories, responder proportions, and baseline correlation structures documented and reported based on the included trials.

While sufficient for trial-level dose-response modeling and power calculations, this method cannot support analyses requiring patient-level interaction terms or subgroup heterogeneity not pre-specified in original trials. However, our LTO validation demonstrates that this limitation does not materially affect predictive accuracy for trial-average treatment effects, which are the primary targets for drug development decision-making. Second, generalizability and transportability of findings require careful consideration given the geographic and demographic composition of source trials. Our evidence base consisted of mainly North American and European adults with mean baseline BMI 31–38 kg/m^2^, age 37–58 years, and 36–68 % female representation. Direct extrapolation to populations with significantly different obesity phenotypes, genetic backgrounds, dietary patterns, or comorbidity profiles is not warranted without adjustment. We implemented transportability methods using arm-normalized Mahalanobis kernel weights to re-calibrate estimates to obesity target population baseline characteristics (BMI around 37 kg/m^2^), but this represents interpolation within the observed covariate space rather than true external validation.

Users applying these models to populations outside the North American/European envelope are warranted and recommended to utilize similar re-weighting methods anchored to local pilot data, or preferably, integrate region-specific Phase II data into updated model fits. Regulatory agencies in non-represented regions may reasonably require local bridging studies prior to extrapolating efficacy and safety conclusions.

Third, nausea-specific adverse event rates for intermediate subcutaneous amycretin doses (2.5, 7.5, 20 mg) were imputed using VINDEL constraints monotone share allocation rather than directly observed, as dose-stratified nausea counts have not been published for these dose levels in Phase Ib/IIa safety reports. VINDEL constraints enforce biological plausibility (monotone dose-response, consistency with observed GI-AE totals, preservation of nausea-to-total-GI ratio ranges from reported doses), but these remain informed estimates pending empirical validation. Sensitivity analyses indicated that plausible uncertainties in imputed nausea rates have minimal impact on primary dose-optimization conclusions (ED50 uncertainty ±0.3 mg, ED80 uncertainty ±0.8 mg) but larger impact on benefit-risk frontier decision boundaries at non-tested intermediate doses (7.5 mg optimal window shifts ±2 mg under alternative imputation scenarios). These imputed values will be replaced with empirical dose-stratified nausea counts when extended safety analyses from ongoing trials become available, and conclusions should be re-evaluated at that time. Users relying on intermediate-dose benefit-risk trade-offs for regulatory submissions should note this caveat and consider sensitivity analyses under alternative nausea allocation scenarios.

Fourth, residual heterogeneity in discontinuation rates (I^2^_DL = 13 %, τ^2^ = 0.03 on logit scale after proper correction), while moderate in magnitude and within acceptable bounds for meta-analysis (I^2^ less than 25 % conventionally classified as “low"), reflects genuine between-trial variation in patient tolerance thresholds, investigator adverse event management strategies, antiemetic co-therapy practices, and supportive care infrastructure quality. Confirmatory Phase III trials may observe discontinuation rates differing from point predictions by ±5–10 % points due to these factors, even at identical doses. Our MAP predictive intervals attempted to quantify this expected trial-to-trial variability, and trial designers should use these interval bounds rather than point estimates when planning discontinuation-contingent adaptive designs, sample size buffers, or futility stopping rules. The predictive intervals incorporate both statistical sampling uncertainty and genuine clinical heterogeneity, providing realistic rather than over-precise planning parameters.

Fifth, our dose-response models assumed classical monotone Emax relationships with Hill coefficient = 1.0 (hyperbolic curves), which represent the most parsimonious functional form consistent with observed data but may not capture more complex phenomena such as, ceiling effects or diminishing returns beyond tested dose ranges (over 60 mg); non-monotonic U-shaped dose-response curves for certain adverse events (such as hypoglycemia showing biphasic patterns in some GLP-1 RAs); long-term tolerance development or tachyphylaxis not observable in trials ≤68 weeks duration; or interaction effects between dose and patient characteristics not identifiable without patient-level data. Extended follow-up beyond one year, supra-maximal dose exploration (over 60 mg), and prospective collection of patient-level interaction data would be needed to test and model these possibilities. Current conclusions should be interpreted as applying to up to 60 mg range over 12–68 week horizons, within the covariate envelope of included trials.

Sixth, while our synthetic target trial emulation framework provides a promising framework of evidence for comparative effectiveness, dose optimization, and trial design planning, it does not replace head-to-head RCTs for definitive regulatory labeling superiority claims. Regulatory acceptance of synthetic comparisons for primary efficacy claims remains jurisdiction-dependent and indication-specific, with proper regulatory guidance continuing to advance. Current precedents suggest synthetic evidence is most accepted for, dose-selection justification in proper regulatory applications; historical control arms in single-arm trials for rare diseases; secondary supportive evidence alongside active-controlled trials; health technology assessment by payers for cost-effectiveness modeling.

Our methods are best positioned to inform Phase IIb/III trial design (sample sizes, dose selection, endpoint timing, adaptive rules), go/no-go portfolio decisions, and secondary evidence synthesis, rather than serving as sole basis for superiority labeling claims. Sponsors should engage regulatory authorities early regarding acceptability of synthetic evidence for their specific development programs and target indications. In addition to that, our predictive models for future trial outcomes should warrant for assumptions about dropout rates that are around 15 % annual, compliance patterns such as dose modification success 85 %, and endpoint measurement variability that may not hold in different trial operational settings.

Multicenter trials ranging from healthcare systems, pragmatic trials with minimal inclusion/exclusion criteria, or trials conducted during public health disruptions may experience different operational characteristics affecting both sample size requirements and achievable statistical power. Trial simulation predictions should be interpreted as applying to well-controlled explanatory efficacy trials conducted under optimal operational conditions, rather than universal predictions invariant to context. Adaptive monitoring with interim data reviews and conditional power re-estimation remains essential even when initial designs follow model-based predictions.

## 5 Conclusions

Synthetic target trial emulation with structured validation demonstrated promising evidence for amylin-pathway development optimization. LTO cross-validation confirmed realistic predictive accuracy (efficacy RMSE: 2.9 % points, calibration slope: 0.61; discontinuation RMSE: 0.18, slope: 1.08), addressing concerns about model overfitting. Bayesian network meta-analysis ranked amycretin 60 mg subcutaneous highest for efficacy (−23.2 % points vs placebo), followed by CagriSema obesity (−17.3 % points), with virtual head-to-head comparisons confirming CagriSema superiority over semaglutide monotherapy (5.4 % points, posterior probability >99.9 %).

Benefit-risk frontier analysis identified an optimal 10–20 mg subcutaneous therapeutic window, balancing ED_80_ efficacy (8.88 mg, 95 % CI: 7.12–11.08) against tolerability thresholds (GI adverse events <75 %, discontinuation <20 %). Heterogeneity quantification through logit-scale correction resolved gastrointestinal adverse event variation completely (I^2^_DL = 0 %, τ^2^ = 0) and reduced discontinuation heterogeneity to moderate levels (I^2^_DL = 13 %, τ^2^ = 0.03), with MAP predictive intervals providing design-ready uncertainty bounds for future trials.

Trial simulations project Phase III confirmatory studies require around 800 to 1,200 participants per arm for 90 % power, with adaptive group sequential designs reducing expected sample sizes by around 15 %. Our work aimed to provide insights for dose selection, population enrichment, and regulatory strategy while quantifying prediction uncertainty. Prospective validation through comparison with completed confirmatory trials will determine real-world alignment of simulated predictions. Generalizability beyond mainly North American/European trial populations requires transportability adjustments or region-specific bridging studies.

## CRediT authorship contribution statement

**Faisal A. Al-Harbi:** Writing – review & editing, Writing – original draft, Visualization, Validation, Resources, Methodology, Investigation, Formal analysis, Data curation, Conceptualization. **Ahmed K. Alsaif:** Writing – review & editing, Writing – original draft, Visualization, Validation, Methodology, Investigation, Formal analysis, Data curation, Conceptualization. **Atheer G. Almutairi:** Writing – review & editing, Writing – original draft, Visualization, Validation, Methodology, Data curation, Conceptualization. **Hussam J. Alshehri:** Writing – review & editing, Writing – original draft, Visualization, Validation, Resources, Data curation, Conceptualization. **Elan A. Aleidan:** Writing – review & editing, Writing – original draft, Visualization, Validation, Data curation, Conceptualization. **Ghaida S. Alabdulaaly:** Writing – review & editing, Writing – original draft, Visualization, Validation, Data curation, Conceptualization. **Mashael E. Alanazi:** Writing – review & editing, Writing – original draft, Visualization, Validation, Supervision. **Ahmed Y. Azzam:** Writing – review & editing, Writing – original draft, Visualization, Validation, Supervision, Project administration, Methodology, Investigation, Formal analysis, Data curation, Conceptualization, Resources, Software.

## Consent to participate

Not applicable.

## Ethical approval

Institutional Review Board approval was not required for this study as it involved analysis of previously published aggregate data from clinical trials without access to individual patient information.

## Data availability statement

All data analyzed in this study were extracted from publicly available published clinical trials as cited in the references. The complete VINDEL (VINe-based DEgree-of-freedom Learning for Synthetic IPD Generation) reproducibility package is publicly available at https://github.com/drazzam/VINDEL.

## Funding

This study received no specific grant from any funding agency in the public, commercial, or not-for-profit sectors.

## References

[1] Hossain M.J., Al-Mamun M., Islam M.R. (2024). Diabetes mellitus, the fastest growing global public health concern: early detection should be focused. Health Sci Rep.

[2] Azzam A.Y., Mora L.M., Morsy M.M., Essibayi M.A., Altschul D.J., Nassar M. (2025). Epidemiological patterns of di patterns of diabetes mellitus in the abetes mellitus in the United States of America: an ob: an observational multicenter analysis from servational multicenter analysis from 1990 to 2024. ASIDE Internal Medicine.

[3] Alfaris N., Waldrop S., Johnson V., Boaventura B., Kendrick K., Stanford F.C. (2024). GLP-1 single, dual, and triple receptor agonists for treating type 2 diabetes and obesity: a narrative review. eClinicalMedicine.

[4] Wang J.Y., Wang Q.W., Yang X.Y., Yang W., Li D.R., Jin J.Y., Zhang H.C., Zhang X.F. (2023). GLP-1 receptor agonists for the treatment of obesity: role as a promising approach. Front Endocrinol.

[5] Goldney J., Hamza M., Surti F., Davies M.J., Papamargaritis D. (2025). Triple agonism based therapies for obesity. Curr Cardiovasc Risk Rep.

[6] Przybyłowski A., Górski M., Gwioździk W., Polaniak R. (2025). Redefining obesity: a narrative review of diagnostic evolution, therapeutic strategies and psychosocial determinants. Healthcare (Basel, Switzerland).

[7] D'Ascanio A.M., Mullally J.A., Frishman W.H. (2024). Cagrilintide: a long-acting amylin analog for the treatment of obesity. Cardiol Rev.

[8] Kruse T., Hansen J., Dahl K., Schäffer L., Sensfuss U., Poulsen C., Schlein M., Hansen A., Jeppesen C., Cour C., Clausen T., Johansson E., Fulle S., Skyggebjerg R., Raun K. (2021). Development of cagrilintide, a long-acting amylin analogue. J Med Chem.

[9] Volčanšek Š., Koceva A., Jensterle M., Janež A., Muzurović E. (2025). Amylin: from mode of action to future clinical potential in diabetes and obesity. Diabetes therapy : research, treatment and education of diabetes and related disorders.

[10] Konnyu K.J., Grimshaw J.M., Trikalinos T.A., Ivers N.M., Moher D., Dahabreh I.J. (2024). Evidence synthesis for complex interventions using meta-regression models. Am J Epidemiol.

[11] Mills E.J., Bansback N., Ghement I., Thorlund K., Kelly S., Puhan M.A., Wright J. (2011). Multiple treatment comparison meta-analyses: a step forward into complexity. Clin Epidemiol.

[12] Hernán M.A., Sauer B.C., Hernández-Díaz S., Platt R., Shrier I. (2016). Specifying a target trial prevents immortal time bias and other self-inflicted injuries in observational analyses. J Clin Epidemiol.

[13] Wang W., Tang W.C., Webster-Clark M., Yu O.H., Filion K.B. (2025). Target trial emulation of cardiovascular outcome trials of medications used to treat type 2 diabetes using real-world data: a systematic review of observational studies. J Clin Epidemiol.

[14] Fu E.L. (2023). Target trial emulation to improve causal inference from observational data: what, why, and how?. J Am Soc Nephrol : JASN (J Am Soc Nephrol).

[15] Hernán M.A., Wang W., Leaf D.E. (2022). Target trial emulation: a framework for causal inference from observational data. JAMA.

[16] Wong K.H.F., Hinchliffe R.J. (2025). Target trial emulation: harnessing real-world data to evaluate surgery and perioperative care interventions. Br J Surg.

[17] Hernán M.A., Dahabreh I.J., Dickerman B.A., Swanson S.A. (2025). The target trial framework for causal inference from observational data: why and when is it helpful?. Ann Intern Med.

[18] Cashin A.G., Hansford H.J., Hernán M.A., Swanson S.A., Lee H., Jones M.D., Dahabreh I.J., Dickerman B.A., Egger M., Garcia-Albeniz X., Golub R.M., Islam N., Lodi S., Moreno-Betancur M., Pearson S.A., Schneeweiss S., Sharp M.K., Sterne J.A.C., Stuart E.A., McAuley J.H. (2025). Transparent reporting of observational studies emulating a target trial-the TARGET statement. JAMA.

[19] Page M.J., McKenzie J.E., Bossuyt P.M., Boutron I., Hoffmann T.C., Mulrow C.D., Shamseer L., Tetzlaff J.M., Akl E.A., Brennan S.E., Chou R., Glanville J., Grimshaw J.M., Hróbjartsson A., Lalu M.M., Li T., Loder E.W., Mayo-Wilson E., McDonald S., McGuinness L.A., Stewart L.A., Thomas J., Tricco A.C., Welch V.A., Whiting P., Moher D. (2021). Revista espanola de cardiologia (English ed).

[20] Kong Y., Yang H., Nie R., Zhang X., Zuo F., Zhang H., Nian X. (2025). Obesity: pathophysiology and therapeutic interventions. Mol Biomed.

[21] Cook T.M., Sandoval D. (2024). Dual-action obesity drug rewires brain circuits for appetite. Nature.

[22] Park B.G., Kim G.M., Lee H.J., Ryu J.H., Kim D.H., Seong J.Y., Kim S., Park Z.Y., Kim Y.J., Lee J., Kim J.I. (2022). Antiobesity therapeutics with complementary dual-agonist activities at glucagon and glucagon-like peptide 1 receptors. Diabetes Obes Metabol.

[23] Keil A.P., Edwards J.K., Richardson D.B., Naimi A.I., Cole S.R. (2014). The parametric g-formula for time-to-event data: intuition and a worked example. Epidemiology.

[24] Naimi A.I., Cole S.R., Kennedy E.H. (2017). An introduction to g methods. Int J Epidemiol.

[25] Bartlett J.W., Olarte Parra C., Granger E., Keogh R.H., van Zwet E.W., Daniel R.M. (2025). G-formula with multiple imputation for causal inference with incomplete data. Stat Methods Med Res.

[26] Chiu Y.H., Wen L., McGrath S., Logan R., Dahabreh I.J., Hernán M.A. (2023). Evaluating model specification when using the parametric G-Formula in the presence of censoring. Am J Epidemiol.

[27] Keil A.P., Daza E.J., Engel S.M., Buckley J.P., Edwards J.K. (2018). A Bayesian approach to the g-formula. Stat Methods Med Res.

[28] Sczepaniak J.P., Owens M.L., Shukla H., Perlegos J., Garner W. (2015). Comparability of weight loss reporting after gastric bypass and sleeve gastrectomy using BOLD data 2008-2011. Obes Surg.

[29] Alfadda A.A., Al-Naami M.Y., Masood A., Elawad R., Isnani A., Ahamed S.S., Alfadda N.A. (2021). Long-term weight outcomes after bariatric surgery: a single center Saudi Arabian cohort experience. J Clin Med.

[30] Maciejewski M.L., Arterburn D.E., Van Scoyoc L., Smith V.A., Yancy W.S., Weidenbacher H.J., Livingston E.H., Olsen M.K. (2016). Bariatric surgery and long-term durability of weight loss. JAMA Surg.

[31] Xie W., Johnston S.S., Waggoner J.R., Doshi I.D., Stokes A.C. (2023). Bariatric surgery and weight loss in the short- and long-term: evidence from NHANES 2015-2018. Clin Obes.

[32] Wolfe B.M., Kvach E., Eckel R.H. (2016). Treatment of obesity: weight loss and bariatric surgery. Circ Res.

[33] Lah S., Hocking S.L. (2025). Treatment of obesity: will incretin agonists make bariatric surgery a thing of the past?. Intern Med J.

[34] Kuhre R.E., Ballarín-González B., Brand C.L., Glendorf T., Madsen K.G., Hjøllund K.R., Hogendorf W.F.J., Ipsen D.H., Lundh S., Kruse T., Petersen S.B., Secher A., Vegge A., Raun K. (2025). The effect of amycretin, a unimolecular glucagon-like peptide-1 and amylin receptor agonist, on body weight and metabolic dysfunction in mice and rats. EBioMedicine.

[35] Laferrère B. (2016). Bariatric surgery and obesity: influence on the incretins. Int J Obes Suppl.

[36] Garvey W.T., Blüher M., Osorto Contreras C.K., Davies M.J., Winning Lehmann E., Pietiläinen K.H., Rubino D., Sbraccia P., Wadden T., Zeuthen N., Wilding J.P.H. (2025). Coadministered cagrilintide and semaglutide in adults with overweight or obesity. N Engl J Med.

[37] Davies M.J., Bajaj H.S., Broholm C., Eliasen A., Garvey W.T., le Roux C.W., Lingvay I., Lyndgaard C.B., Rosenstock J., Pedersen S.D. (2025). Cagrilintide-semaglutide in adults with overweight or obesity and type 2 diabetes. N Engl J Med.

[38] Gasiorek A., Heydorn A., Gabery S., Hjerpsted J.B., Kirkeby K., Kruse T., Petersen S.B., Toubro S., Vegge A., Key C. (2025). Safety, tolerability, pharmacokinetics, and pharmacodynamics of the first-in-class GLP-1 and amylin receptor agonist, amycretin: a first-in-human, phase 1, double-blind, randomised, placebo-controlled trial. Lancet (London, England).

[39] Dahl K., Toubro S., Dey S., Duque do Vale R., Flint A., Gasiorek A., Heydorn A., Jastreboff A.M., Key C., Petersen S.B., Vegge A., Adelborg K. (2025). Amycretin, a novel, unimolecular GLP-1 and amylin receptor agonist administered subcutaneously: results from a phase 1b/2a randomised controlled study. Lancet (London, England).

[40] Frias J.P., Deenadayalan S., Erichsen L., Knop F.K., Lingvay I., Macura S., Mathieu C., Pedersen S.D., Davies M. (2023). Efficacy and safety of co-administered once-weekly cagrilintide 2·4 mg with once-weekly semaglutide 2·4 mg in type 2 diabetes: a multicentre, randomised, double-blind, active-controlled, phase 2 trial. Lancet (London, England).

[41] Enebo L.B., Berthelsen K.K., Kankam M., Lund M.T., Rubino D.M., Satylganova A., Lau D.C.W. (2021). Safety, tolerability, pharmacokinetics, and pharmacodynamics of concomitant administration of multiple doses of cagrilintide with semaglutide 2·4 mg for weight management: a randomised, controlled, phase 1b trial. Lancet (London, England).

[42] Lau D.C.W., Erichsen L., Francisco A.M., Satylganova A., le Roux C.W., McGowan B., Pedersen S.D., Pietiläinen K.H., Rubino D., Batterham R.L. (2021). Once-weekly cagrilintide for weight management in people with overweight and obesity: a multicentre, randomised, double-blind, placebo-controlled and active-controlled, dose-finding phase 2 trial. Lancet (London, England).

