# Supplementary material for: Synthetic target trial emulation and predictive modeling of amylin-pathway therapies for obesity and type 2 diabetes
Source: Metabol Open. 2025 Oct 31;28:100414. doi: 10.1016/j.metop.2025.100414 (PMC12621565; doi:10.1016/j.metop.2025.100414)
Supplement: Multimedia component 1 [file mmc1.docx]

**Supplementary Table 1:** Post-Hoc Analyses Results For Type 2 Diabetes Mellitus, Obesity and Cardiometabolic Outcomes.

| **Trial** | **Active Treatment** | **Comparator** | **Number Active/Control** | **Weight Change (%)** | **ETD Weight (95% CI)** | **P-value** | **≥5% Resp (%)** | **≥10% Resp (%)** | **≥15% Resp (%)** | **≥20% Resp (%)** | **≥25% Resp (%)** | **≥30% Resp (%)** | **Week 12 (%)** | **Week 24 (%)** | **Week 32 (%)** | **Week 36 (%)** | **Week 52 (%)** | **HbA1c Change (pp)** | **ETD HbA1c** | **HbA1c ≤6.5% (%)** | **Waist Change (cm)** | **SBP Change (mmHg)** | **Any TEAE (%)** | **GI AE (%)** | **Nausea (%)** | **Vomiting (%)** | **Diarrhea (%)** | **Constipation (%)** | **SAE (%)** | **DC due AE (%)** | **Target Dose (%)** | **ADA Incidence (%)** |
| --- | --- | --- | --- | --- | --- | --- | --- | --- | --- | --- | --- | --- | --- | --- | --- | --- | --- | --- | --- | --- | --- | --- | --- | --- | --- | --- | --- | --- | --- | --- | --- | --- |
| REDEFINE 1 (CagriSema for Overweight or Obesity) (Investigators 2025) | CagriSema 2.4 mg | Placebo | 2108/705 | -20.4/-3.0 | -17.3 (-18.1, -16.6) | <0.001 | 91.9/31.5 | 83.5/14.3 | 70.1/5.2 | 53.6/1.9 | 34.7/0.0 | 19.3/0.0 | -15.5/-2.5 | -19.7/-3.0 | ---/--- | ---/--- | -20.4/-3.0 | -0.40/-0.03 | -0.38 | 87.7/32.2 | -17.5/-4.0 | -9.9/-3.2 | 92.3/82.3 | 79.6/39.9 | 55.0/--- | 26.1/--- | 24.5/--- | 30.7/--- | 9.8/6.1 | 5.9/3.5 | 57.4 | ---/--- |
| REDEFINE 2 (CagriSema in T2D) (Investigators 2025) | CagriSema 2.4 mg | Placebo | 904/302 | -13.7/-3.4 | -10.4 (-11.2, -9.5) | <0.001 | 83.6/30.8 | 65.6/10.3 | 43.8/2.4 | 22.9/0.5 | 6.1/0.0 | 0.0/0.0 | -10.1/-2.3 | -13.0/-3.1 | ---/--- | ---/--- | -13.7/-3.4 | -1.8/-0.4 | -1.4 | 73.5/15.9 | -11.9/-3.6 | -6.5/-2.4 | 90.2/85.4 | 72.5/34.4 | 44.9/--- | 24.2/--- | 24.3/--- | 24.1/--- | 10.4/12.9 | 8.4/3.0 | 61.9 | ---/--- |
| Amycretin Oral (First-in-human) (Investigators 2025) | Amycretin 2×50 mg | Placebo | 16/12 | -13.1/-1.2 | -11.8 (-14.6, -9.0) | --- | ---/--- | ---/--- | ---/--- | ---/--- | ---/--- | ---/--- | -13.1/-1.2 | ---/--- | ---/--- | ---/--- | ---/--- | -0.3/0.0 | -0.3 | ---/--- | ---/--- | ---/--- | 94.0/33.0 | 69.0/50.0 | ---/--- | ---/--- | ---/--- | ---/--- | 0.0/0.0 | 6.0/0.0 | 100.0 | 6.3/--- |
| Amycretin Subcutaneous (Phase 1b/2a) (Investigators 2025) | Amycretin 60 mg | Placebo | 17/5 | -24.3/-1.1 | -23.2 (-30.8, -15.6) | <0.0001 | ---/--- | ---/--- | ---/--- | ---/--- | ---/--- | ---/--- | ---/--- | ---/--- | ---/--- | -24.3/-1.1 | ---/--- | -0.6/-0.1 | -0.5 | ---/--- | ---/--- | ---/--- | 100.0/100.0 | 94.0/80.0 | ---/--- | ---/--- | ---/--- | ---/--- | 0.0/0.0 | 35.0/0.0 | 100.0 | 29.0/--- |
| CagriSema Phase 2 in T2D (Frias et al. 2023) | CagriSema 2.4 mg | Semaglutide 2.4 mg | 31/31 | -15.6/-5.1 | -10.5 (-14.1, -7.0) | <0.0001 | 71.0/14.0 | 44.0/7.0 | 14.0/--- | ---/--- | ---/--- | ---/--- | -10.1/-4.0 | -14.7/-5.2 | -15.6/-5.1 | ---/--- | ---/--- | -2.2/-1.8 | -0.4 | 89.0/69.0 | ---/--- | ---/--- | 68.0/71.0 | 58.0/32.0 | ---/--- | ---/--- | ---/--- | ---/--- | 0.0/0.0 | 6.0/0.0 | --- | ---/--- |
| CagriSema Phase 1b (Enebo et al. 2021) | Cagrilintide 4.5 mg + Semaglutide 2.4 mg | Placebo + Semaglutide 2.4 mg | 11/4 | -15.4/-8.0 | -7.4 (-12.8, -2.1) | --- | ---/--- | ---/--- | ---/--- | ---/--- | ---/--- | ---/--- | ---/--- | ---/--- | ---/--- | ---/--- | ---/--- | -0.3/-0.2 | -0.5 | ---/--- | ---/--- | ---/--- | 97.0/96.0 | 94.0/79.0 | ---/--- | ---/--- | ---/--- | ---/--- | 0.0/0.0 | 0.0/0.0 | 100.0 | 63.6/--- |
| Cagrilintide Phase 2 (Lau et al. 2021) | Cagrilintide 4.5 mg | Placebo | 101/101 | -10.8/-3.0 | -7.8 (-9.4, -6.2) | <0.001 | 88.7/30.9 | 53.5/10.4 | 18.7/2.9 | ---/--- | ---/--- | ---/--- | -7.3/-2.4 | -10.1/-2.8 | ---/--- | ---/--- | ---/--- | -0.1/-0.1 | -0.3 | ---/--- | ---/--- | ---/--- | 88.0/66.0 | 63.0/32.0 | ---/--- | ---/--- | ---/--- | ---/--- | 1.0/--- | ---/--- | --- | 73.2/--- |

***Notes:*** *Data are presented as Active Treatment/Comparator unless otherwise specified. Weight change data represent percent change from baseline at primary endpoint timepoint. Resp, responders; Week X, weight change at week X; ETD, estimated treatment difference; pp, percentage points; DC, discontinued; ADA, anti-drug antibodies.* ***Abbreviations:*** *ADA, anti-drug antibodies; AE, adverse event; CI, confidence interval; DC, discontinued; ETD, estimated treatment difference; GI, gastrointestinal; HbA1c, glycated hemoglobin; pp, percentage points; Resp, responders; SAE, serious adverse event; SBP, systolic blood pressure; TEAE, treatment-emergent adverse event; T2D, type 2 diabetes.*
