# Supplementary material for: Synthetic target trial emulation and predictive modeling of amylin-pathway therapies for obesity and type 2 diabetes
Source: Metabol Open. 2025 Oct 31;28:100414. doi: 10.1016/j.metop.2025.100414 (PMC12621565; doi:10.1016/j.metop.2025.100414)
Supplement: Multimedia component 2 [file mmc2.docx]

**Supplementary Table 2:** Virtual Head-to-Head Comparisons.

| **Comparison ID** | **Treatment 1** | **Trial 1** | **Time 1 (wks)** | **Number₁** | **Weight Change 1 (%)** | **ETD₁ (pp)** | **Treatment 2** | **Trial 2** | **Time 2 (wks)** | **Number₂** | **Weight Change 2 (%)** | **ETD₂ (pp)** | **Indirect ETD (pp)** | **95% CI** | **P-value** | **Cohen's d** | **P(NI -2.5pp) (%)** | **P(NI -3.0pp) (%)** | **P(SUP) (%)** | **Resp ≥5% T1 (%)** | **Resp ≥10% T1 (%)** | **Resp ≥15% T1 (%)** | **Resp ≥20% T1 (%)** | **Resp ≥5% T2 (%)** | **Resp ≥10% T2 (%)** | **Resp ≥15% T2 (%)** | **Time-Match 12w** | **Time-Match 20w** | **Time-Match 36w** | **Time-Match 68w** | **Population Match** |
| --- | --- | --- | --- | --- | --- | --- | --- | --- | --- | --- | --- | --- | --- | --- | --- | --- | --- | --- | --- | --- | --- | --- | --- | --- | --- | --- | --- | --- | --- | --- | --- |
| 1a | CagriSema 2.4mg | REDEFINE-1 (Obesity) | 68 | 2108 | -20.4 | -17.3 | Amycretin SC 60mg | Amycretin SC 1b/2a | 36 | 17 | -24.3 | -23.2 | 5.9 | (-1.7, 13.5) | 0.131 | 0.39 | 1.3 | 0.9 | 6.2 | 91.9 | 83.5 | 70.1 | 53.6 | --- | --- | --- | -15.5 | -15.5 | -20.4 | -20.4 | Both obesity |
| 1b | CagriSema 2.4mg | REDEFINE-1 (Obesity) | 68 | 2108 | -20.4 | -17.3 | Amycretin SC 20mg | Amycretin SC 1b/2a | 36 | 34 | -22.0 | -23.9 | 6.6 | (2.5, 10.7) | 0.002 | 0.44 | 0.1 | 0.0 | 0.1 | 91.9 | 83.5 | 70.1 | 53.6 | --- | --- | --- | -15.5 | -15.5 | -20.4 | -20.4 | Both obesity |
| 1c | CagriSema 2.4mg | REDEFINE-1 (Obesity) | 68 | 2108 | -20.4 | -17.3 | Amycretin Oral 2×50mg | Amycretin Oral 1 | 12 | 16 | -13.1 | -11.8 | -5.5 | (-8.3, -2.7) | <0.001 | 0.37 | 99.7 | 98.5 | 99.9 | 91.9 | 83.5 | 70.1 | 53.6 | --- | --- | --- | -15.5 vs -13.1 | --- | --- | -20.4 | Both obesity |
| 2a | CagriSema 2.4mg | REDEFINE-2 (T2D) | 68 | 904 | -13.7 | -10.4 | CagriSema 2.4mg | CagriSema Phase 2 T2D | 32 | 31 | -15.6 | -10.5 | 0.1 | (-3.4, 3.6) | 0.955 | 0.01 | 92.2 | 95.5 | 47.9 | 83.6 | 65.6 | 43.8 | 22.9 | 71.0 | 44.0 | 14.0 | -10.1 vs -8.5 | -12.3 vs --- | -13.7 vs -15.6 | -13.7 | Both T2D+obesity |
| 3a | CagriSema 2.4mg | REDEFINE-1 (Obesity) | 68 | 2108 | -20.4 | -17.3 | Cagrilintide 4.5mg | Cagrilintide Phase 2 | 26 | 101 | -10.8 | -7.8 | -9.5 | (-11.1, -7.9) | <0.001 | 0.63 | 100.0 | 100.0 | 100.0 | 91.9 | 83.5 | 70.1 | 53.6 | 88.7 | 53.5 | 18.7 | -15.5 vs --- | --- | -20.4 vs --- | -20.4 vs -10.8 | Both obesity |
| 4a | CagriSema 2.4mg | REDEFINE-1 (Obesity) | 68 | 2108 | -20.4 | -17.3 | CagriSema 4.5+Sema2.4 | CagriSema Phase 1b | 20 | 11 | -15.4 | -7.4 | -9.9 | (-12.1, -7.7) | <0.001 | 0.66 | 100.0 | 100.0 | 100.0 | 91.9 | 83.5 | 70.1 | 53.6 | --- | --- | --- | -15.5 vs --- | -15.5 vs -15.4 | --- | -20.4 | Both obesity |
| 5a | CagriSema (Obesity) | REDEFINE-1 | 68 | 2108 | -20.4 | -17.3 | CagriSema (T2D) | REDEFINE-2 | 68 | 904 | -13.7 | -10.4 | -6.9 | (-8.1, -5.7) | <0.001 | 0.46 | 100.0 | 100.0 | 100.0 | 91.9 | 83.5 | 70.1 | 53.6 | 83.6 | 65.6 | 43.8 | -15.5 vs -10.1 | -15.5 vs -12.3 | -20.4 vs -13.7 | -20.4 vs -13.7 | Obesity vs T2D+obesity |
| 6a | Amycretin SC 60mg | Amycretin SC 1b/2a | 36 | 17 | -24.3 | -23.2 | Amycretin SC 20mg | Amycretin SC 1b/2a | 36 | 34 | -22.0 | -23.9 | 0.7 | (-6.8, 8.2) | 0.852 | 0.05 | 63.1 | 68.4 | 42.1 | --- | --- | --- | --- | --- | --- | --- | --- | --- | -24.3 vs -22.0 | --- | Both obesity |
| 7a | Amycretin SC 60mg | Amycretin SC 1b/2a | 36 | 17 | -24.3 | -23.2 | Amycretin Oral 2×50mg | Amycretin Oral 1 | 12 | 16 | -13.1 | -11.8 | -11.4 | (-18.8, -4.0) | 0.003 | 0.76 | 99.8 | 99.5 | 99.2 | --- | --- | --- | --- | --- | --- | --- | --- vs -13.1 | --- | -24.3 | --- | Both obesity |

***Notes:*** *Data are presented as treatment effect difference (ETD) versus placebo in percentage points (pp) with 95% confidence intervals. Non-inferiority margins of -2.5pp and -3.0pp were used for probability calculations. Cohen's d calculated using estimated pooled standard deviation of 15%. Statistical significance set at P-value<0.05.* ***Abbreviations: CI,*** *confidence interval; ETD, effect treatment difference; NI, non-inferiority; NR, not reported; pp, percentage points; SC, subcutaneous; T2D, type 2 diabetes.*
