# Supplementary material for: Synthetic target trial emulation and predictive modeling of amylin-pathway therapies for obesity and type 2 diabetes
Source: Metabol Open. 2025 Oct 31;28:100414. doi: 10.1016/j.metop.2025.100414 (PMC12621565; doi:10.1016/j.metop.2025.100414)
Supplement: Multimedia component 3 [file mmc3.docx]

**Supplementary Table 3:** Dose-Response Modeling and Longitudinal Kinetics Analysis.

| **Trial** | **Compound/Arm** | **Dose (mg)** | **Route** | **Duration (wks)** | **Number** | **Model Type** | **Emax (%) [95% CI]** | **ED50 (mg) [95% CI]** | **ED80 (mg)** | **Hill Coeff** | **R²** | **Adj. R²** | **RMSE (%)** | **Therapeutic Ratio** | **Final Weight Loss (%)** | **Max Velocity (%/wk)** | **Peak Velocity Period** | **Time to 10% (wks)** | **Time to 15% (wks)** | **Plateau Reached** | **Rate Constant (wk⁻¹)** | **Week 0** | **Week 4** | **Week 8** | **Week 12** | **Week 16** | **Week 20** | **Week 24** | **Week 28** | **Week 36** | **Week 40** | **Week 52** | **Week 68** | **Early Velocity** | **Mid Velocity** | **Late Velocity** | **PK Parameter** | **Exposure-Response** | **Dose Proportionality** | **Steady-State (wks)** | **Biomarker Correlations** |
| --- | --- | --- | --- | --- | --- | --- | --- | --- | --- | --- | --- | --- | --- | --- | --- | --- | --- | --- | --- | --- | --- | --- | --- | --- | --- | --- | --- | --- | --- | --- | --- | --- | --- | --- | --- | --- | --- | --- | --- | --- | --- |
| REDEFINE 1 | CagriSema | 2.4 | SC | 68 | 2108 | Exponential plateau | --- | --- | --- | --- | --- | --- | --- | --- | 20.4 | 1.675 | Week 8-12 | 33.5 | 50.3 | No | 0.0289 | 0.0 | -5.6 | -8.8 | -15.5 | -13.4 | -15.5 | -19.7 | -17.7 | -20.4 | -19.1 | -20.4 | -20.4 | 1.400 | 1.050 | 0.000 | --- | --- | --- | Aroura 4-weeks | --- |
| REDEFINE 2 | CagriSema | 2.4 | SC | 68 | 904 | Exponential plateau | --- | --- | --- | --- | --- | --- | --- | --- | 13.7 | 1.600 | Week 0-4 | 49.8 | --- | No | --- | 0.0 | -6.4 | -9.1 | -10.1 | -11.7 | -12.3 | -13.0 | -13.0 | -13.7 | -13.3 | -13.7 | -13.7 | 1.600 | 0.175 | 0.000 | --- | --- | --- | Aroura 4-weeks | --- |
| Amycretin SC | Amycretin | 60.0 | SC | 36 | 17 | Hill/Emax | 24.66 [23.03-26.30] | 2.22 [1.53-3.22] | 8.88 | 1.00 | 0.986 | 0.959 | 0.66 | 16.00 | 24.3 | 2.112 | Week 0-8 | 14.8 | 22.2 | No | --- | 0.0 | --- | -16.9 | --- | -20.9 | --- | -16.2 | -16.2 | -24.3 | --- | --- | --- | 2.112 | 0.587 | 1.013 | AUC: 120533 h·nmol/L | --- | Yes | Aroura 4-weeks | --- |
| Amycretin SC | Amycretin | 20.0 | SC | 36 | 34 | Hill/Emax | 24.66 [23.03-26.30] | 2.22 [1.53-3.22] | 8.88 | 1.00 | 0.986 | 0.959 | 0.66 | 16.00 | 22.0 | 0.617 | Week 12-24 | 16.4 | 24.5 | No | --- | 0.0 | --- | --- | -7.3 | --- | --- | -14.7 | --- | -22.0 | --- | --- | --- | 0.608 | 0.617 | 0.608 | AUC: 120533 h·nmol/L | --- | Yes | Aroura 4-weeks | --- |
| Amycretin SC | Amycretin | 5.0 | SC | 28 | 16 | Hill/Emax | 24.66 [23.03-26.30] | 2.22 [1.53-3.22] | 8.88 | 1.00 | 0.986 | 0.959 | 0.66 | 16.00 | 16.2 | --- | --- | 17.3 | 25.9 | No | --- | 0.0 | --- | --- | --- | --- | --- | --- | -16.2 | --- | --- | --- | --- | --- | --- | --- | AUC: 120533 h·nmol/L | --- | Yes | Aroura 4-weeks | --- |
| Amycretin SC | Amycretin | 1.25 | SC | 20 | 16 | Hill/Emax | 24.66 [23.03-26.30] | 2.22 [1.53-3.22] | 8.88 | 1.00 | 0.986 | 0.959 | 0.66 | 16.00 | 9.7 | --- | --- | --- | --- | No | --- | 0.0 | --- | --- | --- | --- | --- | --- | --- | --- | --- | --- | --- | --- | --- | --- | AUC: 120533 h·nmol/L | --- | Yes | Aroura 4-weeks | --- |
| Amycretin Oral | Amycretin | 100.0 | Oral | 12 | 16 | Linear | --- | --- | --- | --- | --- | --- | --- | --- | 13.1 | --- | --- | --- | --- | No | --- | 0.0 | --- | --- | -13.1 | --- | --- | --- | --- | --- | --- | --- | --- | --- | --- | --- | AUC: 2328 h·nmol/L | -1.17% per 100 h·nmol/L | Yes | Aroura 4-weeks | --- |
| Amycretin Oral | Amycretin | 50.0 | Oral | 12 | 16 | Linear | --- | --- | --- | --- | --- | --- | --- | --- | 10.4 | --- | --- | --- | --- | No | --- | 0.0 | --- | --- | --- | --- | --- | --- | --- | --- | --- | --- | --- | --- | --- | --- | AUC: 2328 h·nmol/L | -1.17% per 100 h·nmol/L | Yes | Aroura 4-weeks | --- |
| Amycretin Oral | Amycretin | 25.0 | Oral | 12 | 16 | Linear | --- | --- | --- | --- | --- | --- | --- | --- | 12.2 | --- | --- | --- | --- | No | --- | 0.0 | --- | --- | --- | --- | --- | --- | --- | --- | --- | --- | --- | --- | --- | --- | AUC: 2328 h·nmol/L | -1.17% per 100 h·nmol/L | Yes | Aroura 4-weeks | --- |
| Cagrilintide Phase 2 | Cagrilintide | 4.5 | SC | 26 | 101 | Dose-finding | --- | --- | --- | --- | --- | --- | --- | --- | 10.8 | 0.850 | Week 0-4 | --- | --- | No | --- | 0.0 | -3.4 | -5.5 | -7.3 | -8.4 | -9.3 | --- | --- | --- | --- | --- | --- | 0.850 | 0.275 | 0.250 | --- | --- | Yes | --- | --- |
| Cagrilintide Phase 2 | Cagrilintide | 2.4 | SC | 26 | 102 | Dose-finding | --- | --- | --- | --- | --- | --- | --- | --- | 9.7 | --- | --- | --- | --- | No | --- | 0.0 | --- | --- | --- | --- | --- | --- | --- | --- | --- | --- | --- | --- | --- | --- | --- | --- | Yes | --- | --- |
| CagriSema Phase 1b | CagriSema | 4.5/2.4 | SC | 20 | 11 | MAD | --- | --- | --- | --- | --- | --- | --- | --- | 15.4 | --- | --- | --- | --- | No | --- | 0.0 | --- | -10.5 | --- | -14.2 | -15.4 | --- | --- | --- | --- | --- | --- | --- | --- | --- | Cagri Cmax: 170 nmol/L | --- | Yes (cagrilintide) | Aroura 4-weeks | --- |
| CagriSema Phase 2 T2D | CagriSema | 2.4 | SC | 32 | 31 | Fixed dose | --- | --- | --- | --- | --- | --- | --- | --- | 15.6 | --- | --- | --- | --- | No | --- | 0.0 | -8.5 | -13.0 | --- | -15.1 | --- | --- | --- | -15.6 | --- | --- | --- | --- | --- | --- | --- | --- | --- | Aroura 4-weeks | Leptin/sLpR ratio differentiation |

***Abbreviations:*** *Adj., adjusted; AUC, area under curve; Cagri, cagrilintide; CI, confidence interval; Coeff, coefficient; Cmax, maximum concentration; ED50, dose producing 50% of maximum effect; ED80, dose producing 80% of maximum effect; Emax, maximum effect; MAD, multiple-ascending dose; RMSE, root mean square error; SC, subcutaneous; sLpR, soluble leptin receptor; wk, week.*
