# Supplementary material for: Synthetic target trial emulation and predictive modeling of amylin-pathway therapies for obesity and type 2 diabetes
Source: Metabol Open. 2025 Oct 31;28:100414. doi: 10.1016/j.metop.2025.100414 (PMC12621565; doi:10.1016/j.metop.2025.100414)
Supplement: Multimedia component 4 [file mmc4.docx]

**Supplementary Table 4:** Confirmatory Trial Simulations.

| **Sim ID** | **Compound** | **Design Type** | **Primary Endpoint** | **Comparator** | **Number per Arm** | **Total Number** | **Effect Estimate** | **Std Error** | **95% CI Lower** | **95% CI Upper** | **Power (%)** | **Expected P-Value** | **Success Criteria** | **Success Prob (%)** | **Duration (wks)** | **Margin/Threshold** | **Design Features** |
| --- | --- | --- | --- | --- | --- | --- | --- | --- | --- | --- | --- | --- | --- | --- | --- | --- | --- |
| **SUPERIORITY DESIGNS:** | | | | | | | | | | | | | | | | | |
| 001 | REDEFINE 1 CagriSema | Superiority | Weight Loss (%) | Placebo | 500 | 1000 | -17.3 | 0.283 | -17.9 | -16.7 | 99.9 | <0.001 | P(Superiority) ≥97.5% | 99.9 | 68 | --- | Standard RCT |
| 002 | REDEFINE 1 CagriSema | Superiority | Weight Loss (%) | Placebo | 1000 | 2000 | -17.3 | 0.200 | -17.7 | -16.9 | 99.9 | <0.001 | P(Superiority) ≥97.5% | 99.9 | 68 | --- | Standard RCT |
| 003 | REDEFINE 1 CagriSema | Superiority | Weight Loss (%) | Placebo | 1500 | 3000 | -17.3 | 0.163 | -17.6 | -17.0 | 99.9 | <0.001 | P(Superiority) ≥97.5% | 99.9 | 68 | --- | Standard RCT |
| 004 | REDEFINE 2 CagriSema T2D | Superiority | Weight Loss (%) | Placebo | 500 | 1000 | -10.4 | 0.297 | -11.0 | -9.8 | 99.9 | <0.001 | P(Superiority) ≥97.5% | 99.9 | 68 | --- | Standard RCT |
| 005 | REDEFINE 2 CagriSema T2D | Superiority | Weight Loss (%) | Placebo | 1000 | 2000 | -10.4 | 0.210 | -10.8 | -10.0 | 99.9 | <0.001 | P(Superiority) ≥97.5% | 99.9 | 68 | --- | Standard RCT |
| 006 | REDEFINE 2 CagriSema T2D | Superiority | Weight Loss (%) | Placebo | 1500 | 3000 | -10.4 | 0.172 | -10.7 | -10.1 | 99.9 | <0.001 | P(Superiority) ≥97.5% | 99.9 | 68 | --- | Standard RCT |
| 007 | REDEFINE 2 CagriSema T2D | Superiority | HbA1c Change (pp) | Placebo | 500 | 1000 | -1.4 | 0.057 | -1.51 | -1.29 | 99.9 | <0.001 | P(Superiority) ≥97.5% | 99.9 | 68 | --- | Standard RCT |
| 008 | REDEFINE 2 CagriSema T2D | Superiority | HbA1c Change (pp) | Placebo | 1000 | 2000 | -1.4 | 0.040 | -1.48 | -1.32 | 99.9 | <0.001 | P(Superiority) ≥97.5% | 99.9 | 68 | --- | Standard RCT |
| 009 | Amycretin SC 60mg | Superiority | Weight Loss (%) | Placebo | 500 | 1000 | -23.2 | 0.673 | -24.5 | -21.9 | 99.9 | <0.001 | P(Superiority) ≥97.5% | 99.9 | 36 | --- | Standard RCT |
| 010 | Amycretin SC 60mg | Superiority | Weight Loss (%) | Placebo | 1000 | 2000 | -23.2 | 0.476 | -24.1 | -22.3 | 99.9 | <0.001 | P(Superiority) ≥97.5% | 99.9 | 36 | --- | Standard RCT |
| 011 | Amycretin SC 60mg | Superiority | Weight Loss (%) | Placebo | 1500 | 3000 | -23.2 | 0.389 | -24.0 | -22.4 | 99.9 | <0.001 | P(Superiority) ≥97.5% | 99.9 | 36 | --- | Standard RCT |
| 012 | Amycretin Oral 2×50mg | Superiority | Weight Loss (%) | Placebo | 500 | 1000 | -11.8 | 0.849 | -13.5 | -10.1 | 99.9 | <0.001 | P(Superiority) ≥97.5% | 99.9 | 12 | --- | Standard RCT |
| 013 | Amycretin Oral 2×50mg | Superiority | Weight Loss (%) | Placebo | 1000 | 2000 | -11.8 | 0.600 | -13.0 | -10.6 | 99.9 | <0.001 | P(Superiority) ≥97.5% | 99.9 | 12 | --- | Standard RCT |
| 014 | Amycretin Oral 2×50mg | Superiority | Weight Loss (%) | Placebo | 1500 | 3000 | -11.8 | 0.490 | -12.8 | -10.8 | 99.9 | <0.001 | P(Superiority) ≥97.5% | 99.9 | 12 | --- | Standard RCT |
| **NON-INFERIORITY DESIGNS:** | | | | | | | | | | | | | | | | | |
| 015 | CagriSema 2.4 mg | Non-Inferiority | Weight Loss (%) | Semaglutide 2.4 mg | 600 | 1200 | -5.5 | 0.367 | -6.2 | -4.8 | 99.9 | <0.025 | P(NI 2.0pp) ≥95% | 99.9 | 68 | 2.0pp NI margin | Active-controlled NI |
| 016 | CagriSema 2.4 mg | Non-Inferiority | Weight Loss (%) | Semaglutide 2.4 mg | 600 | 1200 | -5.5 | 0.367 | -6.2 | -4.8 | 99.9 | <0.025 | P(NI 3.0pp) ≥95% | 99.9 | 68 | 3.0pp NI margin | Active-controlled NI |
| 017 | CagriSema 2.4 mg | Non-Inferiority | Weight Loss (%) | Semaglutide 2.4 mg | 800 | 1600 | -5.5 | 0.318 | -6.1 | -4.9 | 99.9 | <0.025 | P(NI 2.0pp) ≥95% | 99.9 | 68 | 2.0pp NI margin | Active-controlled NI |
| 018 | CagriSema 2.4 mg | Non-Inferiority | Weight Loss (%) | Semaglutide 2.4 mg | 800 | 1600 | -5.5 | 0.318 | -6.1 | -4.9 | 99.9 | <0.025 | P(NI 3.0pp) ≥95% | 99.9 | 68 | 3.0pp NI margin | Active-controlled NI |
| 019 | CagriSema 2.4 mg | Non-Inferiority | Weight Loss (%) | Semaglutide 2.4 mg | 600 | 1200 | -10.5 | 0.367 | -11.2 | -9.8 | 99.9 | <0.025 | P(NI 2.0pp) ≥95% | 99.9 | 32 | 2.0pp NI margin | Active-controlled NI |
| 020 | CagriSema 2.4 mg | Non-Inferiority | Weight Loss (%) | Semaglutide 2.4 mg | 600 | 1200 | -10.5 | 0.367 | -11.2 | -9.8 | 99.9 | <0.025 | P(NI 3.0pp) ≥95% | 99.9 | 32 | 3.0pp NI margin | Active-controlled NI |
| 021 | CagriSema 2.4 mg | Non-Inferiority | Weight Loss (%) | Semaglutide 2.4 mg | 800 | 1600 | -10.5 | 0.318 | -11.1 | -9.9 | 99.9 | <0.025 | P(NI 2.0pp) ≥95% | 99.9 | 32 | 2.0pp NI margin | Active-controlled NI |
| 022 | CagriSema 2.4 mg | Non-Inferiority | Weight Loss (%) | Semaglutide 2.4 mg | 800 | 1600 | -10.5 | 0.318 | -11.1 | -9.9 | 99.9 | <0.025 | P(NI 3.0pp) ≥95% | 99.9 | 32 | 3.0pp NI margin | Active-controlled NI |
| 023 | Amycretin SC 60 mg | Non-Inferiority | Weight Loss (%) | Amycretin SC 20 mg | 600 | 1200 | -2.3 | 0.367 | -3.0 | -1.6 | 85.2 | <0.025 | P(NI 2.0pp) ≥95% | 85.2 | 36 | 2.0pp NI margin | Active-controlled NI |
| 024 | Amycretin SC 60 mg | Non-Inferiority | Weight Loss (%) | Amycretin SC 20 mg | 600 | 1200 | -2.3 | 0.367 | -3.0 | -1.6 | 94.8 | <0.025 | P(NI 3.0pp) ≥95% | 94.8 | 36 | 3.0pp NI margin | Active-controlled NI |
| 025 | Amycretin SC 60 mg | Non-Inferiority | Weight Loss (%) | Amycretin SC 20 mg | 800 | 1600 | -2.3 | 0.318 | -2.9 | -1.7 | 90.5 | <0.025 | P(NI 2.0pp) ≥95% | 90.5 | 36 | 2.0pp NI margin | Active-controlled NI |
| 026 | Amycretin SC 60 mg | Non-Inferiority | Weight Loss (%) | Amycretin SC 20 mg | 800 | 1600 | -2.3 | 0.318 | -2.9 | -1.7 | 97.2 | <0.025 | P(NI 3.0pp) ≥95% | 97.2 | 36 | 3.0pp NI margin | Active-controlled NI |
| **EQUIVALENCE DESIGNS:** | | | | | | | | | | | | | | | | | |
| 027 | CagriSema 2.4 mg | Equivalence | Weight Loss (%) | Semaglutide 2.4 mg | 800 | 1600 | -5.5 | 0.318 | -6.1 | -4.9 | 65.0 | <0.05 | CI within [±1.5pp] | 65.0 | 68 | ±1.5pp bounds | TOST procedure |
| 028 | CagriSema 2.4 mg | Equivalence | Weight Loss (%) | Semaglutide 2.4 mg | 800 | 1600 | -5.5 | 0.318 | -6.1 | -4.9 | 65.0 | <0.05 | CI within [±2.0pp] | 65.0 | 68 | ±2.0pp bounds | TOST procedure |
| 029 | CagriSema 2.4 mg | Equivalence | Weight Loss (%) | Semaglutide 2.4 mg | 1000 | 2000 | -5.5 | 0.284 | -6.1 | -4.9 | 65.0 | <0.05 | CI within [±1.5pp] | 65.0 | 68 | ±1.5pp bounds | TOST procedure |
| 030 | CagriSema 2.4 mg | Equivalence | Weight Loss (%) | Semaglutide 2.4 mg | 1000 | 2000 | -5.5 | 0.284 | -6.1 | -4.9 | 65.0 | <0.05 | CI within [±2.0pp] | 65.0 | 68 | ±2.0pp bounds | TOST procedure |
| 031 | CagriSema 2.4 mg | Equivalence | Weight Loss (%) | Semaglutide 2.4 mg | 800 | 1600 | -10.5 | 0.318 | -11.1 | -9.9 | 65.0 | <0.05 | CI within [±1.5pp] | 65.0 | 32 | ±1.5pp bounds | TOST procedure |
| 032 | CagriSema 2.4 mg | Equivalence | Weight Loss (%) | Semaglutide 2.4 mg | 800 | 1600 | -10.5 | 0.318 | -11.1 | -9.9 | 65.0 | <0.05 | CI within [±2.0pp] | 65.0 | 32 | ±2.0pp bounds | TOST procedure |
| 033 | CagriSema 2.4 mg | Equivalence | Weight Loss (%) | Semaglutide 2.4 mg | 1000 | 2000 | -10.5 | 0.284 | -11.1 | -9.9 | 65.0 | <0.05 | CI within [±1.5pp] | 65.0 | 32 | ±1.5pp bounds | TOST procedure |
| 034 | CagriSema 2.4 mg | Equivalence | Weight Loss (%) | Semaglutide 2.4 mg | 1000 | 2000 | -10.5 | 0.284 | -11.1 | -9.9 | 65.0 | <0.05 | CI within [±2.0pp] | 65.0 | 32 | ±2.0pp bounds | TOST procedure |
| 035 | Amycretin SC 60 mg | Equivalence | Weight Loss (%) | Amycretin SC 20 mg | 800 | 1600 | -2.3 | 0.318 | -2.9 | -1.7 | 65.0 | <0.05 | CI within [±1.5pp] | 65.0 | 36 | ±1.5pp bounds | TOST procedure |
| 036 | Amycretin SC 60 mg | Equivalence | Weight Loss (%) | Amycretin SC 20 mg | 800 | 1600 | -2.3 | 0.318 | -2.9 | -1.7 | 65.0 | <0.05 | CI within [±2.0pp] | 65.0 | 36 | ±2.0pp bounds | TOST procedure |
| **ADAPTIVE DESIGNS:** | | | | | | | | | | | | | | | | | |
| 037 | REDEFINE 1 CagriSema | Group Sequential | Weight Loss (%) - Interim | Placebo | 400 | 800 | -17.3 | 0.316 | -17.9 | -16.7 | 75.0 | <0.025 | Cross efficacy boundary | 25.0 | 41 | Z ≥ 2.96 | O'Brien-Fleming |
| 038 | REDEFINE 1 CagriSema | Group Sequential | Weight Loss (%) - Final | Placebo | 800 | 1600 | -17.3 | 0.224 | -17.7 | -16.9 | 95.0 | <0.001 | Final efficacy test | 95.0 | 68 | Z ≥ 1.97 | Adjusted α-level |
| 039 | REDEFINE 1 CagriSema | SSR Adaptive | Weight Loss (%) - SSR | Placebo | 1000 | 2000 | -14.7 | 0.179 | -15.1 | -14.3 | 90.0 | <0.001 | Power ≥80% | 90.0 | 68 | 2× max inflation | Conditional power-based |
| 040 | REDEFINE 2 CagriSema T2D | Group Sequential | Weight Loss (%) - Interim | Placebo | 400 | 800 | -10.4 | 0.333 | -11.1 | -9.7 | 75.0 | <0.025 | Cross efficacy boundary | 25.0 | 41 | Z ≥ 2.96 | O'Brien-Fleming |
| 041 | REDEFINE 2 CagriSema T2D | Group Sequential | Weight Loss (%) - Final | Placebo | 800 | 1600 | -10.4 | 0.236 | -10.9 | -9.9 | 95.0 | <0.001 | Final efficacy test | 95.0 | 68 | Z ≥ 1.97 | Adjusted α-level |
| 042 | REDEFINE 2 CagriSema T2D | SSR Adaptive | Weight Loss (%) - SSR | Placebo | 1000 | 2000 | -8.8 | 0.188 | -9.2 | -8.4 | 90.0 | <0.001 | Power ≥80% | 90.0 | 68 | 2× max inflation | Conditional power-based |
| 043 | Amycretin SC 60mg | Group Sequential | Weight Loss (%) - Interim | Placebo | 400 | 800 | -23.2 | 0.671 | -24.5 | -21.9 | 75.0 | <0.025 | Cross efficacy boundary | 25.0 | 22 | Z ≥ 2.96 | O'Brien-Fleming |
| 044 | Amycretin SC 60mg | Group Sequential | Weight Loss (%) - Final | Placebo | 800 | 1600 | -23.2 | 0.474 | -24.2 | -22.2 | 95.0 | <0.001 | Final efficacy test | 95.0 | 36 | Z ≥ 1.97 | Adjusted α-level |
| 045 | Amycretin SC 60mg | SSR Adaptive | Weight Loss (%) - SSR | Placebo | 1000 | 2000 | -19.7 | 0.424 | -20.5 | -18.9 | 90.0 | <0.001 | Power ≥80% | 90.0 | 36 | 2× max inflation | Conditional power-based |

***Abbreviations:*** *CI, confidence interval; HbA1c, glycated hemoglobin; NI, non-inferiority; pp, percentage points; RCT, randomized controlled trial; SSR, sample size re-estimation; TOST, two one-sided tests.*
